# Comparison of the readability of ChatGPT and Bard in medical communication: a meta-analysis

**DOI:** 10.1186/s12911-025-03035-2

**Published:** 2025-09-01

**Authors:** Daphne E. DeTemple, Timo C. Meine

**Affiliations:** 1 Clinic for General, Visceral and Transplant Surgery, Hannover Medical School, Carl-Neuberg-Strasse 1, 30625 Hannover, Germany; 2 PRACTIS Clinician Scientist Program, Dean’s Office for Academic Career Development, Hannover Medical School, Hannover, Germany; 3 Clinic for General, Visceral, Pediatric and Transplantation Surgery, University Hospital RWTH Aachen, Aachen, Germany; 4 Institute for Diagnostic and Interventional Radiology, Hannover Medical School, Carl-Neuberg-Strasse 1, 30625 Hannover, Germany

**Keywords:** Meta-data, Chatbot, Readability, Medicine, Communication

## Abstract

**Background:**

To synthesize the results of various studies on the readability of ChatGPT and Bard in medical communication.

**Methods:**

Systemic literature research was conducted in PubMed, Ovid/Medline, CINAHL, Web-of-Science, Scopus and GoogleScholar to detect relevant publications (inclusion criteria: original research articles, English language, medical topic, ChatGPT-3.5/-4.0, Bard/Gemini, Flesch Reading Ease Score (FRE), Flesch Kincaid Grade Level (FKGL)). Study quality was analyzed using modified Downs-and-Black checklist (max. 8 points), adapted for studies on large language model. Analysis was performed on text simplification and/or text generation with ChatGPT-3.5/-4.0 versus Bard/Gemini. Meta-analysis was conducted, if outcome parameter was reported ≥ 3 studies. In addition, subgroup-analyses among different chatbot versions were performed. Publication bias was analyzed.

**Results:**

Overall, 59 studies with 2342 items were analyzed. Study quality was limited with a mean of 6 points for FRE and 7 points for FKGL. Meta-analysis of text simplification for FRE between ChatGPT-3.5/-4.0 and Bard/Gemini was not significant (mean difference (MD):5.03; 95%-confidence interval (CI):-20.05,30.11; *p* = 0.48). FKGL of simplified texts of ChatGPT-3.5/-4.0 and Bard/Gemini was borderline significant (MD:-1.59; CI:-3.15,-0.04; *p* = 0.05) and subgroup-analysis between ChatGPT-4.0 and Bard was not significant (MD:-1.68; CI:-3.53,0.17; *p* = 0.07). Focused on text acquisition, MD for FRE and FKGL of studies on ChatGPT-3.5/-4.0- and Bard/Gemini-generated texts were significant (MD:-10.36; CI:-13.08,-7.64; *p* < 0.01 / MD:1.62; CI:1.09,2.15; *p* < 0.01). Subgroup-analysis of FRE was significant for ChatGPT-3.5 vs. Bard (MD:-16.07, CI:-24.90,-7.25; *p* < 0.01), ChatGPT-3.5 vs. Gemini (MD:-4.51; CI:-8.73,-0.29: *p* = 0.04), ChatGPT-4.0 vs. Bard (MD:-12.01, CI:-16.22,-7.81; *p* < 0.01) and ChatGPT-4.0 vs. Gemini (MD:-7.91, CI:-11.68,-4.15; *p* < 0.01). Analysis of FKGL in the subgroups was significant for ChatGPT-3.5 vs. Bard (MD:2.85, CI:1.98,3.73; *p* < 0.01), ChatGPT-3.5 vs. Gemini (MD:1.21, CI:0.50,1.93; *p* < 0.01) and ChatGPT-4.0 vs. Gemini (MD:1.95, CI:1.05,2.86; *p* < 0.01), but it was not significant for ChatGPT-4.0 vs. Bard (MD:0.64, CI:-0.46,1.74; *p* = 0.24). Egger’s test was significant in text generation for FRE and FKGL (*p* < 0.01 / *p* < 0.01) and in subgroup ChatGPT-4.0 vs. Bard and ChatGPT-4.0 vs. Gemini (*p* < 0.01 / *p* = 0.02) for FRE as well as in subgroups ChatGPT-3.5 vs. Bard and ChatGPT-4.0 vs. Gemini for FKGL (*p* < 0.01 / *p* < 0.01).

**Conclusion:**

Readability of spontaneously generated texts by Bard/Gemini was slightly superior compared to ChatGPT-3.5/-4.0 and readability of simplified texts by ChatGPT-3.5/-4.0 tended to be improved compared to Bard. Results are limited due study quality and publication bias. Standardized reporting could improve study quality and chatbot development.

**Supplementary Information:**

The online version contains supplementary material available at 10.1186/s12911-025-03035-2.

## Introduction

Since the release of Open-AI’s ChatGPT for use by the general public in the end of 2022 [1], the boundaries of possible applications of artificial intelligence (AI)-based large language models (LLM) in the generation as well as customization of written text are explored. Google released its own competing generative AI “Bard” (now renamed: Gemini) as extension to Google search in 2023 for general use [2]. Since the LLM behind these chatbots are trained on certain data sets and/ or access free content on the internet, the generation of profound and informative texts should be possible. Further, LLM are trained to simulate human conversation, thereby enabling an adaption of the complexity and formality of the language used. In medicine, the possibility to generate texts of a certain reading level or adjust the readability of existing texts has come into focus as potential application to improve communication in clinical, research and educational applications [3]. The question, how patient and educational material should be devised in order to ensure sufficient comprehension of an illness by patients and by students/ junior doctors cannot be answered at the moment. Several authors have tried to assess the quality of content and readability of texts generated and/ or simplified by AI to evaluate a potential application in medicine [4–6]. Different topics and various chatbots have been analyzed in different fields, e.g. internal medicine, surgery, radiology, anesthesiology, pathology or psychology [7]. If clinical appropriateness is given, text readability will have major impact on the comprehension and the compliance of the patient. Recent studies on readability comparisons of ChatGPT- and Bard-responses showed controversial results in medical communication. In one of the first comparisons of AI-generated text, the readability was assessed by two human readers, finding better results for ChatGPT than for Bard [8]. Later, Seth et al. reported better text readability for Bard than for ChatGPT [9], while Kianian et al. have also shown superior text readability for ChatGPT than for Bard [10]. In these studies, text readability has been objectively assessed by the most common measures for evaluation of text complexity based on sentence length and number of syllables: the Flesch Reading Ease Score (FRE) and the Flesch Kincaid Grade Level (FKGL) [11–13]. Depending on the target audience, FRE and FKGL can serve as a guide for enhancing text readability for patients or junior doctors/ medical students. Since controversial results are reported for readability comparisons between different LLM-based chatbots, the AI- or chatbot-algorithm may correspond to the readability of the simplified or generated texts. An overall conclusion on the readability of texts generated by different chatbots has neither been published nor interpreted according the reader’s comprehension level to date. This analysis can be realized by a quantitative synthesis. Thus, the aim of this study is to synthesize the results of published studies on the comparison of readability for ChatGPT- and Bard-generated texts in medical communication using the most common readability scores, FRE and FKGL, in a meta-analysis.

## Methods

### Systematic research of the literature, study inclusion and data extraction

Systematic research of the literature was performed on 16 and 17 January 2025. The databases PubMed, Ovid/Medline, CINAHL, Web-of-Science (including Research Assistant), Scopus (including Scopus AI) and GoogleScholar were searched with the following search term: “(medicine) AND (ChatGPT) AND ((Bard) OR (Gemini)) AND ((flesch reading ease score) OR (flesch kincaid grade level))”. In addition, Web-of-Science Research Assistant and Scopus AI were used with the following prompt: “Find publications on the comparison of ChatGPT and Bard/Gemini using flesch reading ease score or flesch kincaid grade level in medicine! Find as many studies as possible!” There was no restriction of the publication language and date in the systematic literature research. The search-engine, GoogleScholar, was selected to identify additional publications and potential gray literature outside electronic databases. Zotero reference manager was applied (https://www.zotero.org/). Details and complete search strategy are given in the Supplements.

Studies were included when the following inclusion criteria for the meta-analysis were fulfilled: original article, English language, reporting of basic versions of ChatGPT (3.5 and 4.0) and Bard/Gemini, indication of FRE and/ or FKGL in medical context. Exclusion criteria were languages other than English, single chatbot study/ no chatbot comparison, different chatbot versions (ChatGPT-4o/ mini/ Plus, Gemini Pro/ Ultra/ Advanced), no digital access, different readability analysis, no medicine, insufficient outcome data (missing values), abstract, preprint/ non-peer-review publication, duplication, review/ letter/ correspondence/ supplements. If more than one study reported on identical data, the most comprehensive study was selected for study inclusion. Identification of studies was conducted independently and blinded by both investigators (D.E.D. and T.C.M.) and a group consensus was reached for study inclusion. Both investigators (D.E.D. and T.C.M.) documented reasons for study exclusion.

Data on first author, year of publication, country, study design, chatbot (version), medical field, task (acquisition or simplification) and the defined outcome parameters (FRE, FKGL) were extracted from the included studies following a standardized data extraction form. The outcome parameters, FRE and FKGL, were standardized readability measures calculated on average sentence length and average syllables per word. The common formulas to calculate FRE and FKGL of texts are given below with the total number (n) of words and syllables as published by Rouhi et al. [14]:

$$\eqalign{ F\!R\!E{\rm{ }} = & {\rm{ }}\ 206.835{\rm{ }}-{\rm{ }}1.015 \cr &{\rm{ }} \times {\rm{ }}\left( {{n_{words}}/{\rm{ }}{n_{sentences}}} \right){\rm{ }}-{\rm{ }}84.6 \cr &{\rm{ }} \times {\rm{ }}\left( {{n_{syllables}}/{\rm{ }}{n_{words}}} \right) \cr} $$

$$\eqalign{{\rm{FKGL }} \ & {\rm{ = 0}}{\rm{.39 }} \times {\rm{ }}\left( {{{\rm{n}}_{{\rm{words}}}}{\rm{/ }}{{\rm{n}}_{{\rm{sentences}}}}} \right){\rm{ }} \cr &{\rm{ + 11}}{\rm{.8 \ \times }}\left( {{{\rm{n}}_{{\rm{syllables}}}}{\rm{/ }}{{\rm{n}}_{{\rm{words}}}}} \right){\rm{ - 15}}{\rm{.59}} \cr} $$

Although small variations in the calculation of FRE and FKGL were described, FRE and FKGL values were not significantly different between different calculators [15]. Moreover, both readability measures FRE and FKGL were evaluated for the corresponding comprehension capacity of a person at a certain school grade level [11–13]. For both outcome parameters, cohort size, mean and standard deviation were extracted. When a study reported multiple chatbot versions or calculators or tasks, the most appropriate data were extracted where possible (latest chatbot version and calculator average). If necessary, data were converted as described by Hozo et al. (standard deviation = range / 6) and Wan et al. (approximation method in excel spread sheet) [16, 17]. Data were extracted and converted by one investigator (T.C.M.) and given to the second investigator for verification (D.E.D.). When disagreements occurred, a group consensus was reached.

### Study quality assessment

The study quality of the included studies was analyzed with the Downs-and-Black checklist (DBC) modified by Zadro et al. [18], adapted for studies on LLM (LLM-DBC). The original DBC consists of 27 items and is suitable for randomized and non-randomized studies of health care interventions [19]. Focused on our study, we applied LLM-DBC with 8 items and a maximum score of 8 points. Both investigators (D.E.D. and T.C.M.) assessed the study quality independently and a group consensus was reached in case of disagreements for both outcome parameters, FRE and FKGL. Values are given as mean ± standard deviation. Details of the checklist, LLM-DBC, are given in the Supplements.

### Statistical meta-analysis

A narrative analysis was performed if the outcome parameter was reported in less than 3 studies. When the outcome parameter was reported in three or more studies, study heterogeneity was analyzed on a visual approach with forest plots and via statistical analysis with I^2^, Q-statistic and p-value. Heterogeneity was graded with I^2^ as limited (I^2^: 0–40%), moderate (I^2^: 40– 60%), substantial (I^2^: 60–80%) or considerable (I^2^: 80– 100%) as published by Uhlig et al. [20]. When heterogeneity (I^2^ > 40%) and/ or differences in the queries or LLM-based chatbot versions were present, a random-effects model, the Restricted Maximum Likelihood (REML) method with Knapp-Hartung error adjustment, was conducted. In case of limited heterogeneity and uniform study conduction, a fixed-effects model was selected. Thereafter, an overall meta-analysis was performed with a mean difference (MD) as outcome effect between studies on the comparison of ChatGPT-3.5/-4.0- and Bard/Gemini-simplified and/or -generated text. Additionally, subgroup-analyses were conducted among studies with different chatbot versions (ChatGPT-3.5, ChatGPT-4.0, Bard and Gemini). The reason for the subgroup-analyses was that not only different algorithms between different chatbots but also algorithm alterations within a certain chatbot could impact the results. If at least ten studies or datasets were included, funnel plots and Egger’s test would have been used to analyze publication bias. P-values in this study were two-sided and level of significance was < 0.05. All analyses were performed with SPSS (SPSS Statistics, Version 29, IBM, New York). Mean difference (MD), 95% confidence intervals (CI) and p-values are shown.

## Results

### Systematic research of the literature, study inclusion and data extraction

**Fig. 1:**
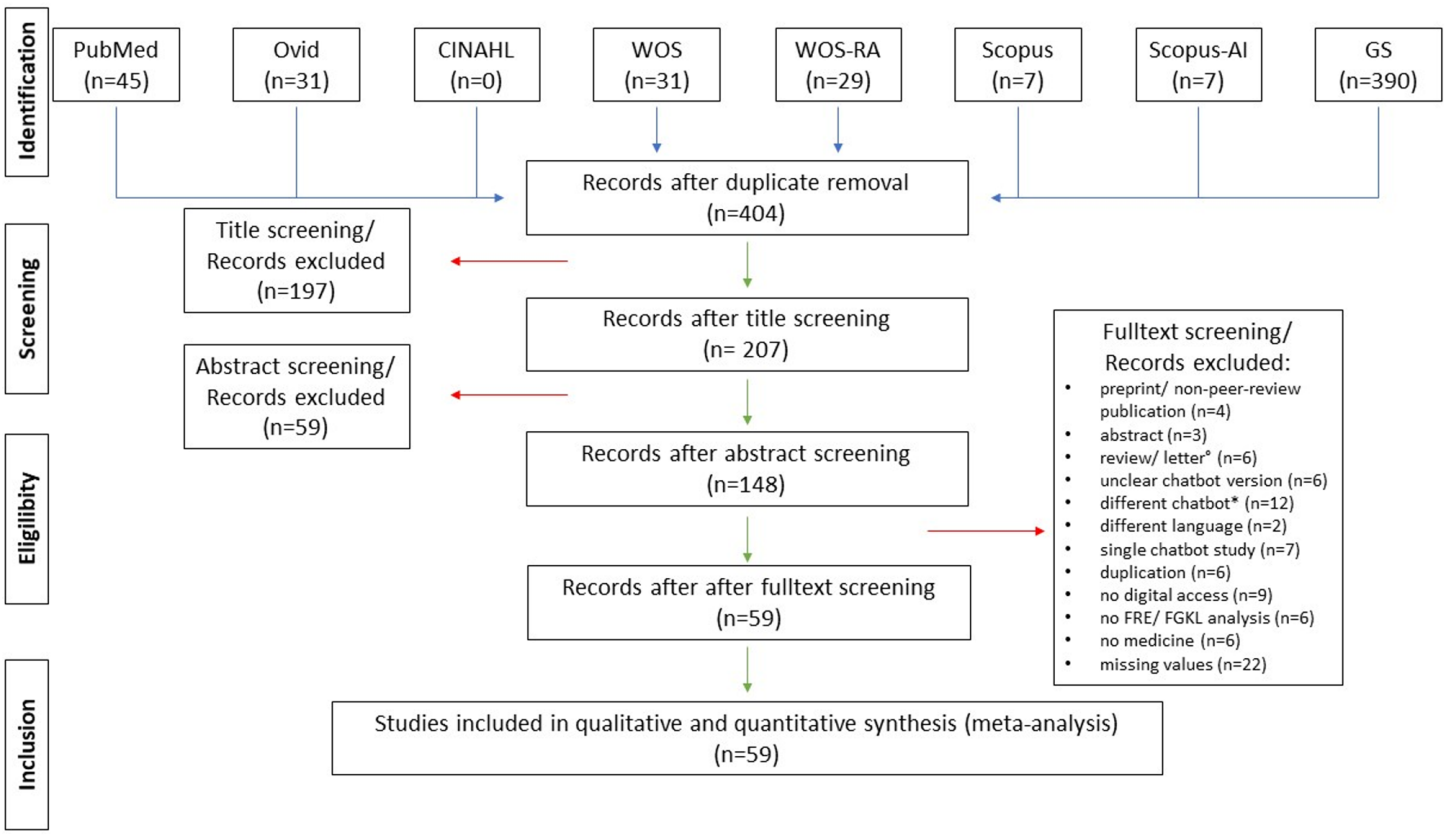
The flow chart visualizes the in- and exclusion of studies adapted from the PRISMA statement (Preferred Reporting Items for Systematic Reviews and Meta-Analysis). Abbreviations: GS = GoogleScholar, n = number, WOS = Web-of-Science, WOS-RA = Web-of-Science-Research-Assistant. °In addition to letters and reviews, reporting of conferences and supplements are excluded. *Different chatbots are ChatGPT-4o/ mini/ Plus, Gemini Pro/ Ultra/ Advanced.

Overall, 540 records could be identified in PubMed, Ovid/Medline, CINAHL, Web-of-Science (including Research Assistant), Scopus (including Scopus AI) and GoogleScholar. After doublet exclusion, 404 records were title-screened. Out of 404 records, we found 207 records on the relevant topic. After abstract and/ or full text analysis, 59 studies fulfilled the inclusion criteria and were incorporated in the meta-analysis [9, 10, 14, 15, 21–23, 24–38, 39–58, 59–75]. The diagram in Fig. 1 visualizes the study in- and exclusion process in adaption to the PRISMA statement (Preferred Reporting Items for Systematic Reviews and Meta-Analysis) (https://www.prisma-statement.org/). Overall, a total number of 2342 items were included given in 78 datasets and 59 studies [9, 10, 14, 15, 21–23, 24–38, 39–58, 59–75]. Text simplification was analyzed in 7 studies [10, 14, 31–33, 65, 66] and text acquisition was analyzed 57 studies and 70 datasets [9, 10, 14, 15, 21–61, 63–65, 67–75]. The medical field varied among the studies with topics ranging from surgery to internal medicine. The majority of the studies were designed to evaluate patient material, but Xie et al. reported medical education for students and junior doctors [71]. Chatbot versions differed among the studies with the following versions applied: ChatGPT-3.5, ChatGPT-4.0, Bard and Gemini. Of note, a few studies analyzed text acquisition and/ or text simplification in several datasets within a single study, which required separate inclusion in this meta-analysis (e.g. Dihan2024b_gen_A/ B/ sim). Details are given in Table 1.

**Table 1:** Study data of the included studies

FirstauthorYEAR_dataset	A-3	Study	Task	Field	ChatGPT_version	Bard_version
Acharya2024	USA	re	generation	NEPH	ChatGPT40	Bard
Adithya2024	IND	re	generation	EMERGENCY	ChatGPT40	Gemini
Alasker2024	SAU	re	generation	URO	ChatGPT40	Bard
Arca2024	TUR	re	generation	CAR/ANE	ChatGPT35	Gemini
Arora2024	IND	re	generation	NER	ChatGPT35	Gemini
Aydın2024	TUR	re	generation	OPH	ChatGPT40	Gemini
Azzopardi2024	GBR	re	generation	OPH	ChatGPT40	Bard
Cao2024	USA	re	generation	RAD	ChatGPT35	Gemini
Carlson2024	USA	re	generation	URO	ChatGPT35	Bard
Connors2024	USA	re	generation	URO	ChatGPT40	Bard
Dihan2024b_gen_A	USA	re	generation	NER	ChatGPT40	Bard
Dihan2024b_gen_B	USA	re	generation	NER	ChatGPT40	Bard
Dihan2024b_sim	USA	re	simplification	NER	ChatGPT40	Bard
Dihan2024c_gen_A	USA	re	generation	PED	ChatGPT40	Bard
Dihan2024c_gen_B	USA	re	generation	PED	ChatGPT40	Bard
Dihan2024c_sim_A	USA	re	simplification	PED	ChatGPT40	Bard
Dihan2024c_sim_B	USA	re	simplification	PED	ChatGPT40	Bard
Dihan2024d_gen_A	USA	re	generation	OPH	ChatGPT40	Bard
Dihan2024d_gen_B	USA	re	generation	OPH	ChatGPT40	Bard
Dihan2024d_sim	USA	re	simplification	OPH	ChatGPT40	Bard
Dogan2024	TUR	re	generation	OPH	ChatGPT35	Bard
ELSenbawy2025	IND	re	generation	SUR	ChatGPT35	Gemini
Garg2024_gen_A	USA	re	generation	ENT	ChatGPT35	Bard
Garg2024_gen_B	USA	re	generation	ENT	ChatGPT35	Bard
Garg2024_gen_C	USA	re	generation	ENT	ChatGPT35	Bard
Garg2024_gen_D	USA	re	generation	ENT	ChatGPT35	Bard
Giammanco2025	USA	re	generation	SUR	ChatGPT40	Gemini
Gunesli2024	TUR	re	generation	END	ChatGPT40	Gemini
Guven2024	TUR	re	generation	SUR	ChatGPT40	Gemini
Hancı2024	TUR	re	generation	ANE	ChatGPT35	Gemini
Ichhpujani2024	IND	re	generation	OPH	ChatGPT35	Bard
Joseph2024	HTI	re	generation	SUR	ChatGPT35	Gemini
Karnan2024	IND	re	generation	RAD	ChatGPT35	Gemini
Kattih2024	USA	re	generation	INT	ChatGPT35	Bard
Kianian2023_gen_A	USA	re	generation	OPH	ChatGPT40	Bard
Kianian2023_gen_B	USA	re	generation	OPH	ChatGPT40	Bard
Kianian2023_sim	USA	re	simplification	OPH	ChatGPT40	Bard
Lauck2024	USA	re	generation	DERM	ChatGPT35	Bard
Lee2024	USA	re	generation	OPH	ChatGPT35	Gemini
Lee2024b	USA	re	generation	CAR	ChatGPT35	Gemini
Lee2024c	CAN	re	generation	SUR	ChatGPT40	Bard
Lee2025z	USA	re	generation	ANE	ChatGPT35	Bard
Li2025	CHN	re	generation	INT	ChatGPT40	Gemini
Lim2023	AUS	re	generation	PRS	ChatGPT40	Bard
Lim2024	AUS	re	generation	PRS	ChatGPT35	Gemini
Lim2024b	AUS	re	generation	PRS	ChatGPT40	Bard
Lim2024c	AUS	re	generation	PRS	ChatGPT40	Gemini
Meyer2024	USA	re	generation	PRS	ChatGPT40	Gemini
Nian2025	USA	re	generation	SUR	ChatGPT40	Gemini
Nian2025b	USA	re	generation	SUR	ChatGPT40	Gemini
Nichani2024	CAN	re	generation	OPH	ChatGPT35	Bard
Ocakoglu2023	TUR	re	generation	GYN	ChatGPT40	Bard
Patnaik2024	USA	re	generation	ANE	ChatGPT35	Bard
Reyhan2024	TUR	re	generation	OPH	ChatGPT40	Gemini
Rocha-Silva2025	BRA	re	generation	NER	ChatGPT40	Gemini
Rokhshad2025	USA	re	generation	DEN	ChatGPT40	Bard
Rouhi2024	USA	re	simplification	SUR	ChatGPT35	Bard
San2024_gen_A	TUR	re	generation	NUC	ChatGPT40	Bard
San2024_gen_B	TUR	re	generation	NUC	ChatGPT40	Bard
San2024_gen_C	TUR	re	generation	NUC	ChatGPT40	Bard
San2024_gen_D	TUR	re	generation	NUC	ChatGPT40	Bard
Seth2023	AUS	re	generation	PRS	ChatGPT35	Bard
Sonmezoglu2024	TUR	re	generation	OPH	ChatGPT35	Gemini
Srinivasan2024_gen	USA	re	generation	SUR	ChatGPT40	Bard
Srinivasan2024_sim	USA	re	simplification	SUR	ChatGPT40	Bard
Tepe2024	TUR	re	simplification	RAD	ChatGPT40	Bard
Tepe2024a	TUR	re	generation	RAD	ChatGPT40	Gemini
Warren2024_gen_A	USA	re	generation	URO	ChatGPT35	Bard
Warren2024_gen_B	USA	re	generation	URO	ChatGPT35	Bard
Warren2025_gen_A	USA	re	generation	URO	ChatGPT35	Bard
Warren2025_gen_B	USA	re	generation	URO	ChatGPT35	Bard
Warren2025_gen_C	USA	re	generation	URO	ChatGPT35	Bard
Wu2024	USA	re	generation	OPH	ChatGPT35	Bard
Xie2023	AUS	re	generation	SUR	ChatGPT40	Bard
Yalla2024	USA	re	generation	OPH	ChatGPT40	Bard
Yau2024	USA	re	generation	EMERGENCY	ChatGPT35	Bard
Yilmaz2024	TUR	re	generation	OPH	ChatGPT35	Bard
Zhao2024	CHN	re	generation	OPH	ChatGPT40	Gemini

Abbreviations: a-3 = alpha-3-code, ANE = anesthesiology, AUS = Australia, BRA = Brazil, CAN = Canada, CAR = cardiology, ChatGPT35 = ChatGPT-3.5, ChatGPT40 = ChatGPT-4.0, CHN = China, CV = cardiovascular medicine, DEN = dentistry, DERM = dermatology, END = endocrinology, ENT = ear-nose-throat, GBR = Great Britain, GYN = gynecology, HTI = Haiti, IND = India, INT = internal medicine, NEPH = nephrology, NER = neurology, NUC = nuclear medicine, OPH = ophthalmology, PED = pediatrics, POL = Poland, PRS = plastic and reconstructive surgery, RAD = radiology, re = retrospective, SAU = Saudi Arabia, SUR = surgery, TUR = turkey, USA = United States of America, URO = urology

### Study quality assessment

The overall quality of the studies was 6 ± 1 points for FRE (n = 46 datasets) and 7 ± 1 points for FKGL (*n* = 74 datasets) according LLM-DBC and in analogy to the modified DBC [18]. Although both mean scores were in the upper level of LLM-DBC, most of the studies were limited in appropriate reporting of statistical analysis. Data are given in Tables 2 and 3.

**Table 2:** Study quality for FRE of the included studies

FirstauthorYEAR_dataset	Task	LLM-DBC/ FRE - sum	1. Is the objective of the study clear?	2. Are the main outcomes clearly described in the Introduction or Methods?	3. Are characteristics of the LLM & questions included in the study clearly described?	4. Are the main findings of the study clearly described?	5. Were LLM & questions designed to participate in the study representative of the entire population from which they were recruited?	6. Were those LLM & questions who were prepared to participate representative and/ or relevant for this research field and study?	7. Were the statistical tests used to assess main outcomes appropriate?	8. Were main outcome measures used accurate (valid and reliable)?
Acharya2024	generation									
Adithya2024	generation	7	1	1	1	1	1	1	0	1
Alasker2024	generation	5	0	1	0	1	0	1	1	1
Arca2024	generation	6	1	1	1	1	0	1	0	1
Arora2024	generation	7	1	1	1	1	1	1	0	1
Aydın2024	generation	6	1	1	0	1	0	1	1	1
Azzopardi2024	generation									
Cao2024	generation	7	1	1	1	1	1	1	0	1
Carlson2024	generation	6	1	1	0	1	0	1	1	1
Connors2024	generation	6	1	1	1	1	1	0	0	1
Dihan2024b_gen_A	generation									
Dihan2024b_gen_B	generation									
Dihan2024b_sim	simplification									
Dihan2024c_gen_A	generation									
Dihan2024c_gen_B	generation									
Dihan2024c_sim_A	simplification									
Dihan2024c_sim_B	simplification									
Dihan2024d_gen_A	generation									
Dihan2024c_sim_B	simplification									
Dihan2024d_gen_B	generation									
Dihan2024d_sim	simplification									
Dogan2024	generation	6	1	1	0	1	0	1	1	1
ELSenbawy2025	generation	7	1	1	1	1	1	1	0	1
Garg2024_gen_A	generation									
Garg2024_gen_B	generation									
Garg2024_gen_C	generation									
Garg2024_gen_D	generation									
Giammanco2025	generation									
Gunesli2024	generation	8	1	1	1	1	1	1	1	1
Guven2024	generation	6	1	1	0	1	0	1	1	1
Hancı2024	generation	6	1	1	0	1	0	1	1	1
Ichhpujani2024	generation	7	1	1	1	1	1	1	0	1
Joseph2024	generation	7	1	1	1	1	1	1	0	1
Karnan2024	generation	7	1	1	1	1	1	1	0	1
Kattih2024	generation	3	1	1	0	0	0	1	0	0
Kianian2023_gen_A	generation									
Kianian2023_gen_B	generation									
Kianian2023_sim	simplification									
Lauck2024	generation	8	1	1	1	1	1	1	1	1
Lee2024	generation	7	1	1	1	1	0	1	1	1
Lee2024b	generation									
Lee2024c	generation	8	1	1	1	1	1	1	1	1
Lee2025z	generation	7	1	1	1	1	1	1	0	1
Li2025	generation									
Lim2023	generation	7	1	1	1	1	1	1	0	1
Lim2024	generation	7	1	1	1	1	1	1	0	1
Lim2024b	generation	7	1	1	1	1	1	1	0	1
Lim2024c	generation	7	1	1	1	1	1	1	0	1
Meyer2024	generation	5	1	1	0	1	0	1	0	1
Nian2025	generation	8	1	1	1	1	1	1	1	1
Nian2025b	generation	8	1	1	1	1	1	1	1	1
Nichani2024	generation	7	1	1	1	1	1	1	0	1
Ocakoglu2023	generation									
Patnaik2024	generation	7	1	1	1	1	1	1	0	1
Reyhan2024	generation	8	1	1	1	1	1	1	1	1
Rocha-Silva2025	generation	8	1	1	1	1	1	1	1	1
Rokhshad2025	generation	7	1	1	1	1	1	1	0	1
Rouhi2024	simplification	7	1	1	0	1	1	1	1	1
San2024_gen_A	generation	8	1	1	1	1	1	1	1	1
San2024_gen_B	generation	8	1	1	1	1	1	1	1	1
San2024_gen_C	generation	8	1	1	1	1	1	1	1	1
San2024_gen_D	generation	8	1	1	1	1	1	1	1	1
Seth2023	generation	6	1	1	0	1	1	1	0	1
Sonmezoglu2024	generation	8	1	1	1	1	1	1	1	1
Srinivasan2024_gen	generation	8	1	1	1	1	1	1	1	1
Srinivasan2024_sim	simplification	8	1	1	1	1	1	1	1	1
Tepe2024	simplification	4	1	1	0	1	0	0	0	1
Tepe2024a	generation	7	1	1	1	1	1	1	0	1
Warren2024_gen_A	generation									
Warren2024_gen_B	generation									
Warren2025_gen_A	generation									
Warren2025_gen_B	generation									
Wu2024	generation									
Xie2023	generation	7	1	1	1	1	1	1	0	1
Yalla2024	generation									
Yau2024	generation									
Yilmaz2024	generation	7	1	1	1	1	1	1	0	1
Zhao2024	generation									

Abbreviations: FRE = Flesch Reading Ease Score, LLM = large language model, LLM-DBC = Adaption of modified Downs-and-Black checklist by Zadro et al. for large language model studies

**Table 3:** Study quality for FKGL of the included studies

FirstauthorYEAR_dataset	Task	LLM-DBC/ FKGL - sum	1. Is the objective of the study clear?	2. Are the main outcomes clearly described in the Introduction or Methods?	3. Are characteristics of the LLM & questions included in the study clearly described?	4. Are the main findings of the study clearly described?	5. Were LLM & questions who were designed to participate in the study representative of the entire population from which they were recruited?	6. Were those LLM & questions who were prepared to participate representative and/ or relevant for this research field and study?	7. Were the statistical tests used to assess main outcomes appropriate?	8. Were main outcome measures used accurate (valid and reliable)?
Acharya2024	generation	7	1	1	1	1	1	1	0	1
Adithya2024	generation	7	1	1	1	1	1	1	0	1
Alasker2024	generation	5	0	1	0	1	0	1	1	1
Arca2024	generation	6	1	1	1	1	0	1	0	1
Arora2024	generation	7	1	1	1	1	1	1	0	1
Aydın2024	generation	6	1	1	0	1	0	1	1	1
Azzopardi2024	generation	6	1	1	0	1	0	1	1	1
Cao2024	generation	7	1	1	1	1	1	1	0	1
Carlson2024	generation	6	1	1	0	1	0	1	1	1
Connors2024	generation									
Dihan2024b_gen_A	generation	7	1	1	1	1	1	1	0	1
Dihan2024b_gen_B	generation	7	1	1	1	1	1	1	0	1
Dihan2024b_sim	simplification	7	1	1	1	1	1	1	0	1
Dihan2024c_gen_A	generation	7	1	1	1	1	1	1	0	1
Dihan2024c_gen_B	generation	7	1	1	1	1	1	1	0	1
Dihan2024c_sim_A	simplification	7	1	1	1	1	1	1	0	1
Dihan2024c_sim_B	simplification	7	1	1	1	1	1	1	0	1
Dihan2024d_gen_A	generation	8	1	1	1	1	1	1	1	1
Dihan2024d_gen_B	generation	8	1	1	1	1	1	1	1	1
Dihan2024d_sim	simplification	8	1	1	1	1	1	1	1	1
Dogan2024	generation									
ELSenbawy2025	generation	7	1	1	1	1	1	1	0	1
Garg2024_gen_A	generation	8	1	1	1	1	1	1	1	1
Garg2024_gen_B	generation	8	1	1	1	1	1	1	1	1
Garg2024_gen_C	generation	8	1	1	1	1	1	1	1	1
Garg2024_gen_D	generation	8	1	1	1	1	1	1	1	1
Giammanco2025	generation	6	1	0	1	1	1	1	0	1
Gunesli2024	generation	8	1	1	1	1	1	1	1	1
Guven2024	generation	6	1	1	0	1	0	1	1	1
Hancı2024	generation	6	1	1	0	1	0	1	1	1
Ichhpujani2024	generation	7	1	1	1	1	1	1	0	1
Joseph2024	generation	7	1	1	1	1	1	1	0	1
Karnan2024	generation	7	1	1	1	1	1	1	0	1
Kattih2024	generation	3	1	1	0	0	0	1	0	0
Kianian2023_gen_A	generation	7	1	1	1	1	1	1	0	1
Kianian2023_gen_B	generation	7	1	1	1	1	1	1	0	1
Kianian2023_sim	simplification	5	1	1	1	1	0	0	0	1
Lauck2024	generation	8	1	1	1	1	1	1	1	1
Lee2024	generation	7	1	1	1	1	0	1	1	1
Lee2024b	generation	4	1	1	0	1	0	0	0	1
Lee2024c	generation	8	1	1	1	1	1	1	1	1
Lee2025z	generation	7	1	1	1	1	1	1	0	1
Li2025	generation	7	1	1	1	1	1	1	0	1
Lim2023	generation	7	1	1	1	1	1	1	0	1
Lim2024	generation	7	1	1	1	1	1	1	0	1
Lim2024b	generation	7	1	1	1	1	1	1	0	1
Lim2024c	generation	7	1	1	1	1	1	1	0	1
Meyer2024	generation									
Nian2025	generation	8	1	1	1	1	1	1	1	1
Nian2025b	generation	8	1	1	1	1	1	1	1	1
Nichani2024	generation	7	1	1	1	1	1	1	0	1
Ocakoglu2023	generation	8	1	1	1	1	1	1	1	1
Patnaik2024	generation	7	1	1	1	1	1	1	0	1
Reyhan2024	generation	8	1	1	1	1	1	1	1	1
Rocha-Silva2025	generation	8	1	1	1	1	1	1	1	1
Rokhshad2025	generation	7	1	1	1	1	1	1	0	1
Rouhi2024	simplification	7	1	1	0	1	1	1	1	1
San2024_gen_A	generation	8	1	1	1	1	1	1	1	1
San2024_gen_B	generation	8	1	1	1	1	1	1	1	1
San2024_gen_C	generation	8	1	1	1	1	1	1	1	1
San2024_gen_D	generation	8	1	1	1	1	1	1	1	1
Seth2023	generation	6	1	1	0	1	1	1	0	1
Sonmezoglu2024	generation	8	1	1	1	1	1	1	1	1
Srinivasan2024_gen	generation	8	1	1	1	1	1	1	1	1
Srinivasan2024_sim	simplification	8	1	1	1	1	1	1	1	1
Tepe2024	simplification	4	1	1	0	1	0	0	0	1
Tepe2024a	generation	7	1	1	1	1	1	1	0	1
Warren2024_gen_A	generation	7	1	1	1	1	1	1	0	1
Warren2024_gen_B	generation	7	1	1	1	1	1	1	0	1
Warren2025_gen_A	generation	7	1	1	1	1	1	1	0	1
Warren2025_gen_B	generation	7	1	1	1	1	1	1	0	1
Warren2025_gen_C	generation	7	1	1	1	1	1	1	0	1
Wu2024	generation	7	1	1	1	1	1	1	0	1
Xie2023	generation	7	1	1	1	1	1	1	0	1
Yalla2024	generation	8	1	1	1	1	1	1	1	1
Yau2024	generation	7	1	1	1	1	1	1	0	1
Yilmaz2024	generation									
Zhao2024	generation	8	1	1	1	1	1	1	1	1

Abbreviations: FKGL = Flesch Kincaid Grade Level, LLM = large language model, LLM-DBC = Adaption of modified Downs-and-Black checklist by Zadro et al. for large language model studies

### Statistical meta-analysis

#### Text simplification

Text simplification and readability analysis with FRE and FGKL was only reported in 7 studies. Heterogeneity was present in study design among all studies and additional analysis revealed I^2^ of 0.98 for FRE and I^2^ of 0.97 for FKGL. Thus, REML model was applied for meta-analysis. MD of text simplification for FRE between ChatGPT-3.5/-4.0 and Bard/Gemini were not significantly different (MD: 5.03; CI: -20.05, 30.11; *p* = 0.48).

**Fig. 2:**
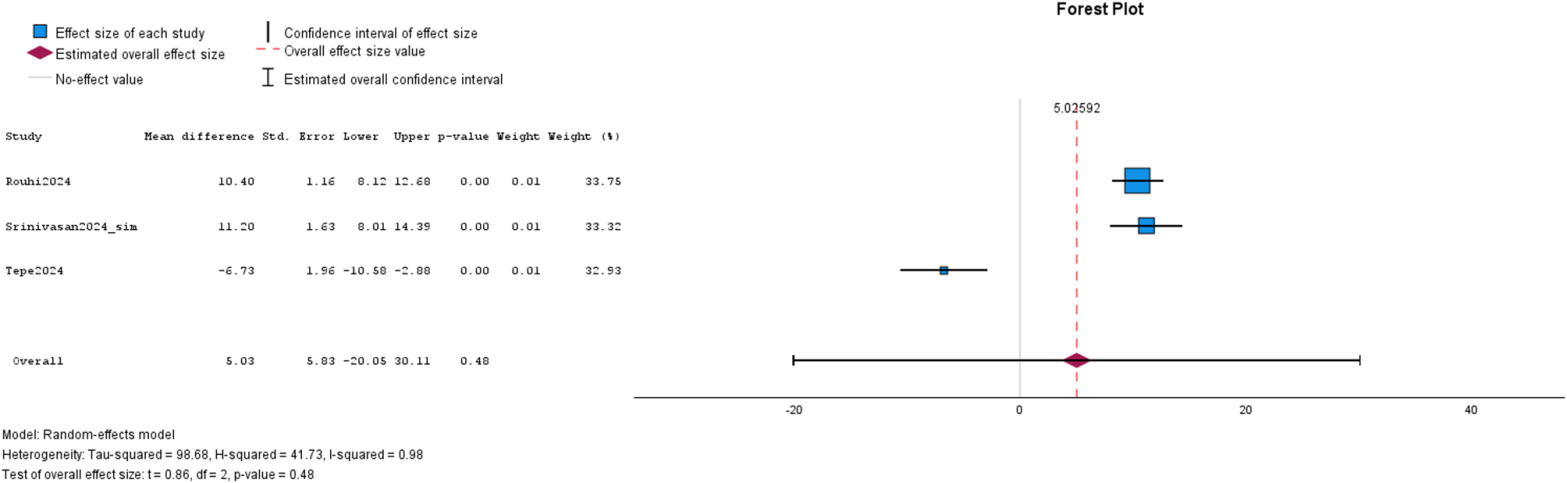
The forest plot for FRE in text simplification of the studies on the comparison of ChatGPT and Bard/Gemini is visualized. The effect sizes of the studies are represented by blue or green boxes and corresponding confidence intervals are visualized with black bars. Estimated overall effect size is depicted with the violet rhombus and estimated overall confidence interval is shown with whiskers. The overall effect size value is given as dashed red line. Studies are labeled with first author and year of publication. Abbreviations: ChatGPT35 = ChatGPT-3.5 and ChatGPT40 = ChatGPT-4.0.

**Fig. 3:**
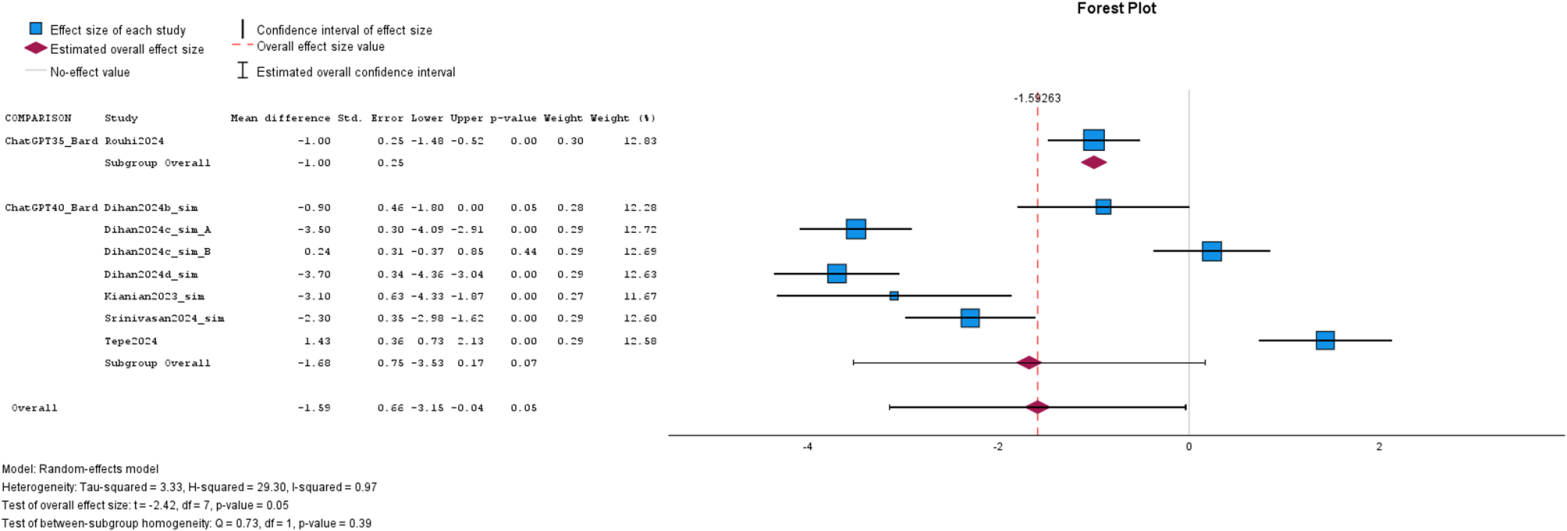
The forest plot for FKGL in text simplification of the studies on the comparison of ChatGPT and Bard/Gemini is visualized. The effect sizes of the studies are represented by blue or green boxes and corresponding confidence intervals are visualized with black bars. Estimated overall effect size is depicted with the violet rhombus and estimated overall confidence interval is shown with whiskers. The overall effect size value is given as dashed red line. Studies are labeled with first author and year of publication. Abbreviations: ChatGPT35 = ChatGPT-3.5 and ChatGPT40 = ChatGPT-4.0.

Subgroup-analysis of text simplification for FRE was not suitable due to the limited number of studies. In a narrative approach, Rouhi et al. and Srinivasan et al. showed a significantly more favorable FRE score for ChatGPT as compared to Bard [14, 65]. In contrast, Tepe et al. reported more favorable FRE for Bard than ChatGPT-4.0 [66]. Focused on FKGL of LLM-simplified text, there was a borderline significant difference between ChatGPT-3.5/-4.0 and Bard (MD: -1.59, CI: -3.15, -0.04; *p* = 0.05). Subgroup-analysis of text simplification for FKGL between ChatGPT-4.0 and Bard was not significant (MD: -1.68, CI: -3.53, 0.17; *p* = 0.07). Of note, no included study reported on the comparison of ChatGPT-3.5/4.0 and Gemini. Details are given in Figs. 2 and 3; Tables 4 and 5.

**Table 4:** Data on FRE of the included studies

FirstauthorYEAR_dataset	Task	Comparison	ChatGPT_FRE			Bard_FRE		
			*n*	mean	sd	*n*	mean	sd
Acharya2024	generation	ChatGPT40_Bard						
Adithya2024	generation	ChatGPT40_Gemini	5	41.88	16.64	5	50.62	12.71
Alasker2024	generation	ChatGPT40_Bard	52	39.83^a^	9.45^a^	52	53.63^a^	10.82^a^
Arca2024	generation	ChatGPT35_Gemini	100	38.66^a^	11.58^a^	100	43.86^a^	14.45^a^
Arora2024	generation	ChatGPT35_Gemini	3	26.57	10.58	3	48.3	6.56
Aydın2024	generation	ChatGPT40_Gemini	40	31.79	26.72^c^	40	37.39	7.98^c^
Azzopardi2024	generation	ChatGPT40_Bard						
Cao2024	generation	ChatGPT35_Gemini	60	29	8	60	30	8
Carlson2024	generation	ChatGPT35_Bard	15	30.4	8.01	15	49.67	9.61
Connors2024	generation	ChatGPT40_Bard	32	25.69	9.66	32	49.83	15.68
Dihan2024b_gen_A	generation	ChatGPT40_Bard						
Dihan2024b_gen_B	generation	ChatGPT40_Bard						
Dihan2024b_sim	simplification	ChatGPT40_Bard						
Dihan2024c_gen_A	generation	ChatGPT40_Bard						
Dihan2024c_gen_B	generation	ChatGPT40_Bard						
Dihan2024c_sim_A	simplification	ChatGPT40_Bard						
Dihan2024c_sim_B	simplification	ChatGPT40_Bard						
Dihan2024d_gen_A	generation	ChatGPT40_Bard						
Dihan2024d_gen_B	generation	ChatGPT40_Bard						
Dihan2024d_sim	simplification	ChatGPT40_Bard						
Dogan2024	generation	ChatGPT35_Bard	114	26.1	7.3	114	29.2	10.7
ELSenbawy2025	generation	ChatGPT35_Gemini	3	37.13	23.17	3	39.5	4.75
Garg2024_gen_A	generation	ChatGPT35_Bard						
Garg2024_gen_B	generation	ChatGPT35_Bard						
Garg2024_gen_C	generation	ChatGPT35_Bard						
Garg2024_gen_D	generation	ChatGPT35_Bard						
Giammanco2025	generation	ChatGPT40_Gemini						
Gunesli2024	generation	ChatGPT40_Gemini	44	21.5	11.2	44	36.7	10.2
Guven2024	generation	ChatGPT40_Gemini	59	46.42	9.3	59	51.91	8.02
Hancı2024	generation	ChatGPT35_Gemini	100	21.31^a^	6.79^a^	100	23.53^a^	7.89^a^
Ichhpujani2024	generation	ChatGPT35_Bard	25	22.51	7.04	25	57.91	7.1
Joseph2024	generation	ChatGPT35_Gemini	3	33.27	8.28	3	35.43	11.26
Karnan2024	generation	ChatGPT35_Gemini	3	36.3	7.01	3	46.77	4.96
Kattih2024	generation	ChatGPT35_Bard	7	58.5	8.06	7	50.1	5.7
Kianian2023_gen_A	generation	ChatGPT40_Bard						
Kianian2023_gen_B	generation	ChatGP40_Bard						
Kianian2023_sim	simplification	ChatGPT40_Bard						
Lauck2024	generation	ChatGPT35_Bard	12	40.9	6.9	12	60.6	5.3
Lee2024	generation	ChatGPT35_Gemini	50	40.17	3.92	50	39.47	11.31
Lee2024b	generation	ChatGPT35_Gemini						
Lee2024c	generation	ChatGPT40_Bard	36	21.68	2.78	36	42.89	4.03
Lee2025z	generation	ChatGPT35_Bard	20	35.5	9.62	20	52.15	8.79
Li2025	generation	ChatGPT40_Gemini						
Lim2023	generation	ChatGPT40_Bard	5	38.28	10.41	5	43.44	6.25
Lim2024	generation	ChatGPT35_Gemini	15	35.22	7.44	15	35.86	9.81
Lim2024b	generation	ChatGPT40_Bard	6	24.7	6.1	6	34.1	4.8
Lim2024c	generation	ChatGPT40_Gemini	10	43.39	12.52^c^	10	39.41	8.23^c^
Meyer2024	generation	ChatGPT40_Gemini	10	33.3	12.5	10	37.5	11.5
Nian2025	generation	ChatGPT40_Gemini	9	25.8^c^	6.1^c^	9	36.8^c^	9.9^c^
Nian2025b	generation	ChatGPT40_Gemini	13	28.4c	9.3c	13	38.5c	8.8c
Nichani2024	generation	ChatGPT35_Bard	11	32.9	20.31	11	49.7	12.47
Ocakoglu2023	generation	ChatGPT40_Bard						
Patnaik2024	generation	ChatGPT35_Bard	33	30	21.2	33	55.4	11.6
Reyhan2024	generation	ChatGPT40_Gemini	20	28.85	8.44	20	34.7	8.79
Rocha-Silva2025	generation	ChatGPT40_Bard	14	35.46	9.58	14	38.94	11.93
Rokhshad2025	generation	ChatGPT40_Bard	20	45.8	10.8	20	62	7
Rouhi2024	simplification	ChatGPT35_Bard	21	76.9	1.2	21	66.5	5.2
San2024_gen_A	generation	ChatGPT40_Bard	30	25.46	7.76	30	37.3	9.35
San2024_gen_B	generation	ChatGPT40_Bard	29	25.35	7.41	29	32.91	10.17
San2024_gen_C	generation	ChatGPT40_Bard	30	27.45	6.62	30	38.67	11.16
San2024_gen_D	generation	ChatGPT40_Bard	30	26.47	7.11	30	29.4	11.7
Seth2023	generation	ChatGPT35_Bard	6	37.68	12.96	6	47.47	15.32
Sonmezoglu2024	generation	ChatGPT35_Gemini	22	39.9	10.58	22	51.92	7.85
Srinivasan2024_gen	generation	ChatGPT40_Bard	66	42.7	9.7	66	56.3	11.6
Srinivasan2024_sim	simplification	ChatGPT40_Bard	66	74	7.2	66	62.8	11.1
Tepe2024	simplification	ChatGPT40_Bard	30	58.1	7.47	29	64.83	7.61
Tepe2024a	generation	ChatGPT40_Gemini	20	37.15	8.74	20	52.45	9.12
Warren2024_gen_A	generation	ChatGPT35_Bard						
Warren2024_gen_B	generation	ChatGPT35_Bard						
Warren2025_gen_A	generation	ChatGPT35_Bard						
Warren2025_gen_B	generation	ChatGPT35_Bard						
Warren2025_gen_C	generation	ChatGPT35_Bard						
Wu2024	generation	ChatGPT35_Bard						
Xie2023	generation	ChatGPT40_Bard	3	31.2	3.5	3	33.9	5.6
Yalla2024	generation	ChatGPT40_Bard						
Yau2024	generation	ChatGPT35_Bard						
Yilmaz2024	generation	ChatGPT35_Bard	20	34.38	9.75	20	55.5	8.48
Zhao2024	generation	ChatGPT40_Gemini						

Abbreviations: ChatGPT35 = ChatGPT-3.5, ChatGPT40 = ChatGPT-4.0, FRE = Flesch Reading Ease Score. ^a^Data are converted (approximation method; s. Methods). ^c^Data are assumed to represent mean/ standard deviation as presented in the primary study

**Table 5:** Data on FKGL of the included studies

FirstauthorYEARdataset	Task	Comparison	ChatGPT_FKGL			Bard_FKGL		
			*n*	mean	sd	*n*	mean	sd
Acharya2024	generation	ChatGPT40_Bard	35	11.1	1.9	35	11.5	2
Adithya2024	generation	ChatGPT40_Gemini	5	9.12	2.77	5	8.84	1.92
Alasker2024	generation	ChatGPT40_Bard	52	12.53^a^	2.05^a^	52	10.3^a^	1.9^a^
Arca2024	generation	ChatGPT35_Gemini	100	12.31^a^	2.11^a^	100	10.55^a^	1.77^a^
Arora2024	generation	ChatGPT35_Gemini	3	12.27	2.83	3	10.9	4.28
Aydın2024	generation	ChatGPT40_Gemini	40	13.49	3.92^c^	40	11.76	1.35^c^
Azzopardi2024	generation	ChatGPT40_Bard	59	5.75	0.98^b^	59	8.02	1.31^b^
Cao2024	generation	ChatGPT35_Gemini	60	14	2	60	13	2
Carlson2024	generation	ChatGPT35_Bard	15	14.23	1.5	15	10.1	1.56
Connors2024	generation	ChatGPT40_Bard						
Dihan2024b_gen_A	generation	ChatGPT40_Bard	20	8.6	0.6	20	9.4	1.3
Dihan2024b_gen_B	generation	ChatGPT40_Bard	20	5.6	0.7	20	7	0.6
Dihan2024b_sim	simplification	ChatGPT40_Bard	20	5.7	0.8	20	6.6	1.9
Dihan2024c_gen_A	generation	ChatGPT40_Bard	20	8.58	0.6	20	8.25	0.6
Dihan2024c_gen_B	generation	ChatGPT40_Bard	20	4.31	0.7	20	8.01	0.7
Dihan2024c_sim_A	simplification	ChatGPT40_Bard	20	3.76	0.6	20	7.26	1.2
Dihan2024c_sim_B	simplification	ChatGPT40_Bard	10	5.75	0.7	10	5.51	0.7
Dihan2024d_gen_A	generation	ChatGPT40_Bard	20	8.1	0.8	20	7.8	0.5
Dihan2024d_gen_B	generation	ChatGPT40_Bard	20	3.6	0.6	20	6.9	0.6
Dihan2024d_sim	simplification	ChatGPT40_Bard	20	3.7	0.9	20	7.4	1.2
Dogan2024	generation	ChatGPT35_Bard						
ELSenbawy2025	generation	ChatGPT35_Gemini	3	10.97	3.71	3	10.07	0.99
Garg2024_gen_A	generation	ChatGPT35_Bard	28	14.39	1.53	28	9.88	1.34
Garg2024_gen_B	generation	ChatGPT35_Bard	28	13.16	1.87	28	9.94	1.26
Garg2024_gen_C	generation	ChatGPT35_Bard	28	10.54	1.82	28	9.63	1.27
Garg2024_gen_D	generation	ChatGPT35_Bard	28	14.03	1.36	28	10.04	1.65
Giammanco2025	generation	ChatGPT40_Gemini	10	12.5	1.84	10	9.9	1.86
Gunesli2024	generation	ChatGPT40_Gemini	44	15	2.1	44	11.7	1.8
Guven2024	generation	ChatGPT40_Gemini	59	12.13	1.71	59	10.18	1.45
Hancı2024	generation	ChatGPT_Gemini	100	14.54^a^	0.97^a^	100	14.67^a^	1.24^a^
Ichhpujani2024	generation	ChatGPT35_Bard	25	15.4	1.8	25	8.94	1.2
Joseph2024	generation	ChatGPT35_Gemini	3	12.86	2.41	3	10.27	1.25
Karnan2024	generation	ChatGPT35_Gemini	3	12	2.31	3	9.33	0.81
Kattih2024	generation	ChatGPT35_Bard	7	9.3	1.1	7	9.9	0.77
Kianian2023_gen_A	generation	ChatGPT40_Bard	10	6.3	1.2	10	10.5	0.8
Kianian2023_gen_B	generation	ChatGP40_Bard	10	9.2	1.3	10	10.8	0.7
Kianian2023_sim	simplification	ChatGPT40_Bard	9	8	1	9	11.1	1.6
Lauck2024	generation	ChatGPT35_Bard	12	11.9	1.6	12	9	1.1
Lee2024	generation	ChatGPT35_Gemini	50	12.16	0.69	50	12.48	1.86
Lee2024b	generation	ChatGPT35_Gemini	208	15.92	4.58^c^	208	13.5	3.54^c^
Lee2024c	generation	ChatGPT40_Bard	36	14	0.45	36	11	0.57
Lee2025z	generation	ChatGPT35_Bard	20	13.53	1.9	20	10.2	1.73
Li2025	generation	ChatGPT40_Gemini	192	13.19	1.96	192	13.32	2.44
Lim2023	generation	ChatGPT40_Bard	5	12.84	1.2	5	11.98	1.54
Lim2024	generation	ChatGPT35_Gemini	15	13.49	1.35	15	12.22	2.05
Lim2024b	generation	ChatGPT40_Bard	6	12.8	1.1	6	10.8	0.8
Lim2024c	generation	ChatGPT40_Gemini	10	12.26	3.21^c^	10	12.44	1.22^c^
Meyer2024	generation	ChatGPT40_Gemini						
Nian2025	generation	ChatGPT40_Gemini	9	15.9^c^	1.1^c^	9	12.5^c^	1.5^c^
Nian2025b	generation	ChatGPT40_Gemini	13	14.5^c^	1.4^c^	13	12.4^c^	1.8^c^
Nichani2024	generation	ChatGPT35_Bard	11	14.06	4.42	11	9.86	2.4
Ocakoglu2023	generation	ChatGPT40_Bard	15	13.99	1.25	15	9.83	1.2
Patnaik2024	generation	ChatGPT35_Bard	33	14.7	2.7	33	9.4	2
Reyhan2024	generation	ChatGPT40_Gemini	20	14.64	1.7	20	12.46	1.73
Rocha-Silva2025	generation	ChatGPT40_Gemini	14	11.63	1.47	14	12.11	1.8
Rokhshad2025	generation	ChatGPT40_Bard	20	10.1	1.9	20	7.6	1.2
Rouhi2024	simplification	ChatGPT35_Bard	21	5.9	0.8	21	6.9	0.8
San2024_gen_A	generation	ChatGPT40_Bard	30	14.57	1.19	30	11.49	1.59
San2024_gen_B	generation	ChatGPT40_Bard	29	14.65	1.38	29	12.42	1.71
San2024_gen_C	generation	ChatGPT40_Bard	30	14.25	1.1	30	11.35	1.8
San2024_gen_D	generation	ChatGPT40_Bard	30	14.38	1.2	30	13.01	1.97
Seth2023	generation	ChatGPT35_Bard	6	10.15	1.84	6	9.71	3.12
Sonmezoglu2024	generation	ChatGPT35_Gemini	22	12	2.21	22	10.06	1.69
Srinivasan2024_gen	generation	ChatGPT40_Bard	66	11.8	2	66	9.8	2.6
Srinivasan2024_sim	simplification	ChatGPT40_Bard	66	6.2	1.5	66	8.5	2.4
Tepe2024	simplification	ChatGPT40_Bard	30	9	1.6	29	7.57	1.09
Tepe2024a	generation	ChatGPT40_Gemini	20	13.55	1.9	20	9.92	1.69
Warren2024_gen_A	generation	ChatGPT35_Bard	9	14.6	1.8	9	10.8	2.1
Warren2024_gen_B	generation	ChatGPT35_Bard	9	11	1.2	9	9.6	1.4
Warren2025_gen_A	generation	ChatGPT35_Bard	3	12.7	0.3	3	11.2	0.7
Warren2025_gen_C	generation	ChatGPT35_Bard	10	13.4	1.2	10	10.8	2.7
Wu2024	generation	ChatGPT35_Bard	4	12.3	2.4	4	9.7	2.1
Xie2023	generation	ChatGPT40_Bard	3	13.5	0.7	3	13	1.01
Yalla2024	generation	ChatGPT40_Bard	95	13.01	0.65	95	7.9	0.65
Yau2024	generation	ChatGPT35_Bard	10	10.76	1.43	10	8.91	1.75
Yilmaz2024	generation	ChatGPT35_Bard						
Zhao2024	generation	ChatGPT40_Gemini	27	15.07^a^	0.6^a^	27	11.45^a^	0.71^a^

Abbreviations: ChatGPT35 = ChatGPT-3.5, GPT40 = ChatGPT-4.0, FKGL = Flesch Kincaid Grade Level. ^a^ Data are converted (approximation method; s. Methods). ^b^ Data are converted (standard deviation = range / 6; s. Methods). ^c^ Data are assumed to represent mean/ standard deviation as presented in the primary study

Analysis of publication bias was not performed due to the limited number of studies. Additional data are given in the Supplements.

#### Text acquisition

Of the 59 included studies, 57 studies reported FRE and/ or FKGL in text acquisition (s. Table 1). As mentioned before, there was a heterogenous study design (e.g. variation in field and chatbot version). Heterogeneity analysis showed I^2^ of 0.93 for FRE and I^2^ of 0.98 for FKGL among these studies on text acquisition. In REML, MD for FRE and FKGL of ChatGPT-3.5/-4.0- and Bard/Gemini-generated text were significant in the pooled data (MD: -10.36; CI: -13.08, -7.64; *p* < 0.01 / MD: 1.62; CI: 1.09, 2.15; *p* < 0.01). Egger’s test was significant for FRE (*p* < 0.01) and FKGL (*p* < 0.01). Subgroup-analysis of FRE was significant for the comparison of ChatGPT-3.5 vs. Bard (MD: -16.07, CI: -24.90, -7.25; *p* < 0.01), ChatGPT-3.5 vs. Gemini (MD: -4.51; CI: -8.73, -0.29; *p* = 0.04), ChatGPT-4.0 vs. Bard (MD: -12.01, CI: -16.22, -7.81; *p* < 0.01) and ChatGPT-4.0 vs. Gemini (MD: -7.91, CI: -11.68, -4.15; *p* < 0.01). Egger’s test for FRE was not significant in subgroup-analysis ChatGPT-3.5 vs. Bard and ChatGPT-3.5 vs. Gemini (*p* = 0.06 and *p* =0.66), but significant in subgroup-analysis ChatGPT-4.0 vs. Bard and ChatGPT-4.0 vs. Gemini (*p* < 0.01 and *p* = 0.02).

Analysis of FKGL in the subgroups was significant for ChatGPT-3.5 vs. Bard (MD:2.85, CI:1.98, 3.73; *p* < 0.01), ChatGPT-3.5 vs. Gemini (MD:1.21, CI: 0.50, 1.93; *p* < 0.01) and ChatGPT-4.0 vs. Gemini (MD: 1.95, CI: 1.05, 2.86; *p* < 0.01), but it was not significant different in the subgroup ChatGPT-4.0 vs. Bard (MD: 0.64, CI: -0.46, 1.74; *p* = 0.24). Egger’s test for FKGL was significant in subgroup-analysis ChatGPT-3.5 vs. Bard and ChatGPT-4.0 vs. Gemini (*p* < 0.01, *p* < 0.01), but it was not significant in subgroup-analysis ChatGPT-3.5 vs. Gemini and ChatGPT-4.0 vs. Bard (*p* = 0.11, *p* = 0.84).

**Fig. 4:**
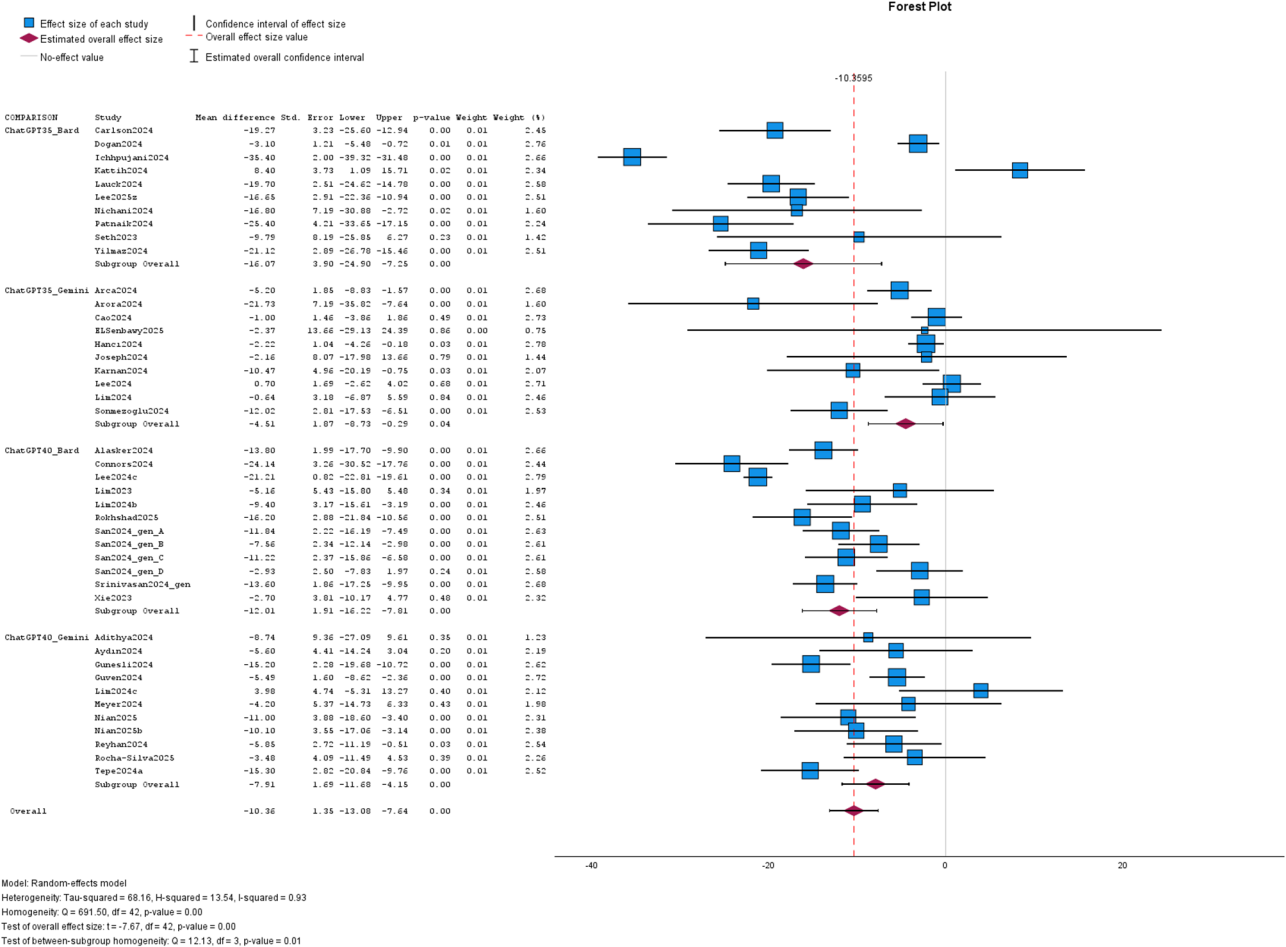
The forest plot illustrates the results for FRE in text acquisition of the studies on the comparison of ChatGPT and Bard/Gemini. The effect sizes of the studies are represented by blue or green boxes and corresponding confidence intervals are visualized with black bars. Estimated overall effect size is depicted with the violet rhombus and estimated overall confidence interval is shown with whiskers. The overall effect size value is given as dashed red line. Studies are labeled with first author and year of publication. Abbreviations: ChatGPT35 = ChatGPT-3.5 and ChatGPT40 = ChatGPT-4.0.

**Fig. 5:**
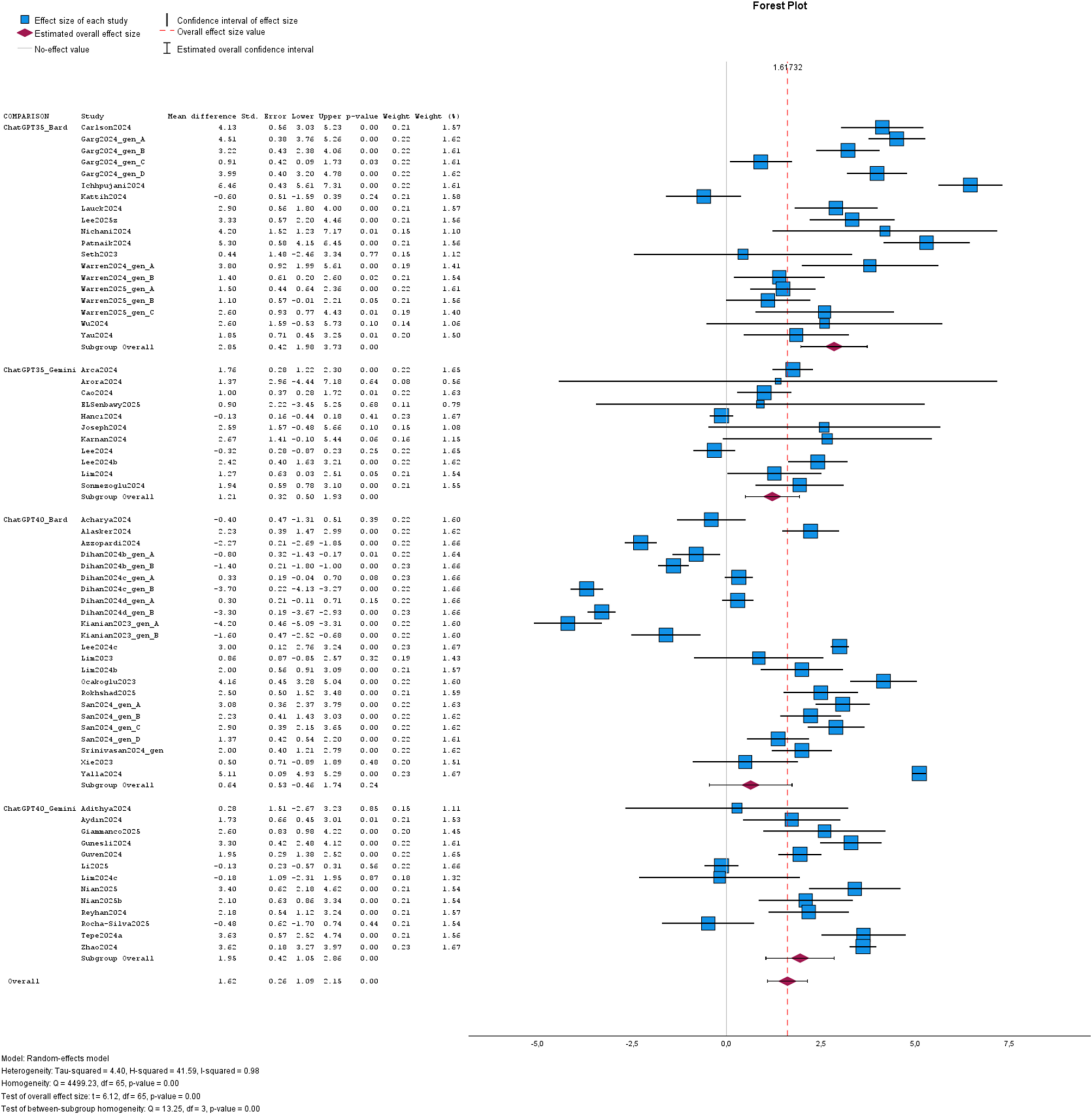
The forest plot illustrates the results for FKGL in text acquisition of the studies on the comparison of ChatGPT and Bard/Gemini. The effect sizes of the studies are represented by blue or green boxes and corresponding confidence intervals are visualized with black bars. Estimated overall effect size is depicted with the violet rhombus and estimated overall confidence interval is shown with whiskers. The overall effect size value is given as dashed red line. Studies are labeled with first author and year of publication. Abbreviations: ChatGPT35 = ChatGPT-3.5 and ChatGPT40 = ChatGPT-4.0

Data are given in Tables 4 and 5 and visualized in Figs. 4 and 5. Additional data are given in the Supplements.

## Discussion

In review of our results, the study quality of all analyzed studies is in the upper range with a mean of 6 and 7 out of 8 points for the readability scores FRE and FKGL according the LLM-DBC and in analogy to the modified DBC [18]. Overall, the study quality is limited due to the feasibility design and appropriate reporting of statistical analysis of the included studies. In addition, no standardized means for reporting on methodology of AI studies or specific instruments to assess the quality of studies on AI exist yet. Standardization of reporting could enhance the quality and reproducibility of studies on the application of LLM. Therefore, general recommendations for the handling of AI in text generation need to be developed by the scientific community to improve both reporting and assessment of studies on AI application. We suggest to use a checklist such as LLM-DBC for standardized reporting and quality assessment of studies on AI-generated text, which is given in the Supplements.

Concerning text simplification, there is no difference in FRE and FKGL between ChatGPT-3.5/-4.0 and Bard/Gemini among seven studies. No subgroup-analysis could be conducted for FRE due to the limited number of studies. However, there is a borderline significance in FKGL in simplified texts of ChatGPT-3.5/-4.0 and Bard with a MD of -1.59. In analogy, subgroup-analysis is almost different with a similar MD of -1.68 for FKGL between ChatGPT-4.0 and Bard, but this is also not statistically significant. This indicates an improved readability of texts simplified by ChatGPT-3.5/-4.0 compared to texts simplified by Bard, also with a difference of about one school grade. An explanation for this potentially improved simplification function of ChatGPT-3.5/-4.0 due to detection and application of the formula behind FKGL is discussed in the literature [10]. Overall, no conclusion on relevant differences between the application of ChatGPT-3.5/-4.0 and Bard/Gemini for the simplification of text in medical communication can be drawn due to limited data.

Meta-analysis of the studies comparing text acquisition using ChatGPT-3.5/-4.0 versus Bard/Gemini results in a significant difference with an overall MD of -10.36 points for FRE. This can be a relevant discrepancy, as 10 points on the FRE scoring scale are commonly set as interval between school grade levels. Higher scores indicate an easier readability than lower scores [76]. The average FRE of texts generated by ChatGPT-3.5/-4.0 is at least 10 points lower than the average FRE of the responses by Bard/Gemini. Hence, Bard/Gemini-acquired text is easier to read than text generated by ChatGPT-3.5/-4.0. The reason might be the difference between the underlying algorithms of the LLM and the pool of data used for training of the chatbots. Since OpenAI and Google give limited information on the AI-algorithms and chatbot systems behind ChatGPT and Bard, it is difficult to identify specific differences that could explain our findings. Bard has been designed as service-oriented tool with the ability to use simple speech, developed on the LaMDA LLM family. It was reportedly trained on 137 billion parameters, and retrieved data online for each query as summarized by Patnaik et al. [59]. In contrast, ChatGPT has been designed to possess natural language understanding and reasoning ability and is based on the multimodal LLM GPT. In recent versions, it was trained on at least 175 billion parameters as argued by Patnaik et al. [59]. In the latest version ChatGPT-4.0, multi-modal input (including images for queries) is incorporated, but ChatGPT’s referencing background is limited as it has no real-time access to the internet when responding to prompts as mentioned in the review by Thirunavukarasu et al. [3]. This seems to be the most striking difference: Although ChatGPT has been trained on more parameters than Bard, it has no real-time internet access. Hence, the superior readability of Bard/Gemini-generated texts as compared to ChatGPT-3.5/-4.0-generated texts using FRE might be explained by the difference of real-time internet access. In accordance to the FRE results, the meta-analysis of the second readability score, FKGL, in text acquisition is also significant with an overall MD of 1.62 points between ChatGPT-3.5/-4.0- and Bard/Gemini-responses. Moreover, readability scores of texts generated by Bard or Gemini are significantly improved compared to texts generated by ChatGPT-3.5 or ChatGPT-4.0 in the subgroup-analyses, with the exception of the subgroup-analysis ChatGPT-4.0 vs. Bard. This implies a general difference between basic versions of ChatGPT and Bard/Gemini, which could rely on the AI-algorithm. As available information on chatbot systems and AI-algorithms are restricted, possible further technical explanations for the different results of applied LLMs are difficult to discuss. However, a lower correctness (“reliability”) for Bard/Gemini-generated text than for ChatGPT-generated text is suggested in the literature [50]. It might be possible that a high accuracy or correctness of responses might be associated with low readability scores (i.e. harder to read). According to OpenAI, GPT-4 reduces hallucinations, responds 82% less frequently to disallowed content and also responds 29% more often to sensitive content such as medical advice when compared to GPT-3.5 [77]. Furthermore, technical improvements have been achieved by leveraging data from prior models, rewriting prompts into new similar boundary prompts, application of rule-based reward models and collecting ranking data from labelers who attempted to circumvent the desired GPT-4-launch behavior [77]. Last, the GPT-4 model has been fine-tuned on human preferences using reinforcement learning from human feedback [77]. All these and further developments may explain improvements in reliability of responses by ChatGPT-4.0. The drawback of an improved reliability of responses might be a lower text readability in comparison to Bard/Gemini or LLM-based chatbots. However, this is a hypothesis that requires to be verified by objective comparison of the response content.

In our point of view, an alignment of text readability and comprehension level of the reader is very important. The “Patient Safety Network of the Agency for Healthcare Research and Quality” recommends a reading level corresponding to the 6th grade at school for the general population. This is given as a FRE of about 80 to 90 points or a FKGL of 6 points [76, 78, 79]. In the studies on ChatGPT- and Bard/Gemini-simplified or -generated texts, mean FRE ranges from 21.31 to 76.9 points and mean FKGL ranges from 3.6 to 15.92 points. Thus, both chatbots have a large deviation in text readability and often do not reach the recommendation by the Patient Safety Network [78]. Focused on text simplification, mean FRE ranges from 58.1 to 76.9 and mean FGKL ranges from 3.7 to 11.1 of simplified texts. Albeit it has been the task for the LLM-based chatbots to simplify the text readability, the recommended reading level for patients is not reached in FRE and tends to be achieved in FKGL only by ChatGPT-3.5/-4.0. Interestingly, Rooney et al. have reported on the readability of 2585 patient educational materials published in high-impact medical journals from 1998 to 2018 [80]. In this study, the patient educational materials provided by humans met the American Medical Association recommendation of the 6th grade level in only 2.1% of cases and the National Institutes of Health recommendation of the 8th grade level in 8.2% [80]. Moreover, the readability does not differ significantly over time in this study [80]. Another recent study by Decker et al. has also found that the readability of surgeon- as well as ChatGPT-3.5-generated information on risks, benefits and therapeutic alternatives is more complex than the 6th grade reading level [4]. Thus, neither human- nor chatbot-generated patient material is well adapted to patients’ comprehension levels. Since LLM-based chatbots are trained on insufficient human-generated patient material, it is conceivable that chatbot-generated patient material is also not sufficient in text readability. In addition, basic chatbot versions are readily available, e.g. ChatGPT-3.5 or 4.0 and Bard/Gemini. This may lead to misinformation or delayed medical consultation and treatment, if medical information generated by old or basic chatbot versions is either too difficult to understand or incorrect and unprecise.

Of course, LLM-based chatbots adapted to the patient’s reading level can support medical communication, which could improve the patient’s comprehension of a disorder and also the patient’s compliance to a treatment. Practical applications of LLM-based chatbots for clinicians are evident, e.g. simplification of patient-facing materials. For example, patient handouts on handling of medical devices (such as inhalation devices, insulin pumps, pacemakers or cardiac assist devices) could be simplified using LLM-based chatbots. Another example is the patient’s informed consent, which is required for every medical intervention, ranging from blood transfusion over radiological examinations to surgical procedures. If, however, the patient consent material used does not ensure sufficient understanding due to incomprehensible langue, the material does not serve its purpose. Based on the above studies by Rooney et al. [80] and Decker et al. [4], this appears to be the current reality. If AI could reliably transform all existing patient material into easier (and maybe different) reading levels, this would greatly benefit patients and clinicians alike. The same consideration applies to medical websites or patient Frequently Asked Questions. Furthermore, some chatbot providers, such as Google’s Gemini and OpenAI’s ChatGPT, offer communication in several languages. With growing need for multi-language information in every day clinical work, the translation of patient material into different languages at a set reading level would be a game-changer in medical communication.

Apart from these linguistic considerations, half of the patients could not accurately recall information provided by clinicians in an ambulatory setting [81]. A reliable chatbot which is accessible around the clock could provide continued support in a patient-friendly language. On the other hand, AI-based chatbots could also support clinicians to extract the relevant information from medical reports for the specific question and patient visit. The development of such electronic health record summaries or EHR summaries generated by AI-models could be crucial for the reduction of screen-time and improve personalized decision making [82–84], especially in complex cases and in an outpatient setting. Another promising therapeutic application of LLM-based chatbots may be the field of psychosomatic and psychological disorders. Communication is often the basis of primary therapy and the demand increasingly exceeds the existing resources. Already in the 1960s, Joseph Weizenbaum, a researcher from Massachusetts Institute of Technology’s Computer Science and Artificial Intelligence Laboratory, published ELIZA, a simple computer program simulating human conversation and emotions [85]. The COVID-19 pandemic boosted the need for alternatives to direct human interaction and furthered the evaluation of artificial intelligence as digital alternative. A 2022 randomized and controlled Chinese study found favorable effects for young adults with depressive symptoms [86]. Already in 2019, a research group investigating the use of chatbots for psychiatric treatment found favorable results for the use of artificial conversational agents [87]. However, in all these applications, chatbots would have to be controlled regularly to ensure no information is lost or misinformation spread. Thus, improvement of LLM-based chatbots is of paramount importance for patient safety. To ensure the safety of AI-based chatbots, collaboration of data scientists and medical experts is required to make AI-models and their output transparent, explainable and interpretable, as recommended in a recent review by Sadeghi et al. [88]. Therefore, developers could provide prompts and responses in a data repository or registry, which could be regularly evaluated by medical experts. Since the readability of human- and AI-generated patient material is insufficient for the patient’s comprehension level, policymakers could also support the development and application of AI-models with population-wide surveys on the readability of such patient material as kind of real-world target-audience evaluation. A certified and free chatbot version for patients and medical doctors might be an ideal amendment in healthcare systems.

Finally, there are several limitations to our study. First, the exclusion of pre-prints, abstracts or perspectives and articles in languages different to English in the systematic literature research may affect the results. Publication types with limited or no peer-review might be an alternative communication in science, especially for negative results, but the research quality is debatable. Second, our readability analysis is only based on FRE and FKGL. Although FRE and FKGL are commonly used, several other readability instruments are available for text evaluation (e.g. SMOG index) and specific assessment techniques can affect the results [89, 90]. Third, no recommended instrument for the assessment of the quality of studies on AI or LLM exists to date in the scientific community. With no standardization for the reporting of readability scores, this differs greatly between the studies (e.g. reporting summary data). Further, the number of studies on the comparison of the readability of specific chatbot versions or LLM as well as on specific readability scores is limited. Thus, heterogeneity and publication bias are present, which limit the evidence of the results. In addition, different versions of chatbots were applied within the included studies resulting in even lower numbers of available studies for the subgroup-analysis. Furthermore, spontaneous responses of chatbots are more commonly analyzed in the literature than specifically generated and simplified text. Albeit text simplification is possible using a target readability score in the prompt, the minority of studies have investigated this valuable application of simplification tasks between different chatbots (e.g. ChatGPT and Bard/Gemini). A further limitation is that several publications on the use of LLM in medical communication with comparison of ChatGPT and Bard/Gemini seem to be published from identical research groups according to the authorship profile. Additionally, the study by Xie et al. was designed for education of medical students and junior doctors [71], whereas all other studies were on patient communication. Another limitation of our study is the focus on readability, because the evaluation of the content with regard to the correctness (“reliability”) of text simplified or generated by LLM is an important issue. Since correctness needs to be assessed by humans and no standardized instrument exists yet (e.g. a recommended Likert-scale), further research with comparable and objective outcome measures is required. Moreover, our findings cannot be generalized on chatbots or LLM due to different technologies. All aspects limit the transferability of the results.

## Conclusion

In our meta-analysis, Bard/Gemini-generated texts scored slightly higher for FRE and slightly lower for FKGL as compared to ChatGPT-3.5/-4.0-generated texts. This difference corresponds to a difference in the reader’s comprehension level of about one school grade level. Albeit there is this significant difference, neither texts generated by ChatGPT-3.5/-4.0 nor texts generated by Bard/Gemini reach the recommended reading levels. When the task for the LLM-based chatbots was set to text simplification, readability of texts generated by ChatGPT-3.5/-4.0 tend to be improved compared to the readability of texts generated by Bard. In analogy, the recommended reading level seems to be better achieved with texts simplified by ChatGPT-3.5/-4.0 than by Bard. The results are limited due to the heterogeneity of the studies, publication bias and limited data on simplification tasks. Whether the readability of simplified texts depends on the combination of human-generated training material, LLM-based chatbot or the prompt with or without input of a targeted readability level, will need further investigation. Another important question will be if LLM-assisted information material improves treatment success and patient outcome or the learning curve of medical students. Open access publication of primary text data, queries and responses could increase the reproducibility and accelerate the development of LLM-based chatbots. Therefore, legal aspects including patient rights, data protection, and how to handle texts generated or augmented by AI need to be determined. Overall, the ongoing development of AI networks will have the most important effect on the readability of generated or simplified text. Standardization of the reporting and outcome measures is required to improve ongoing chatbot development.

## Electronic supplementary material

Below is the link to the electronic supplementary material.

Supplementary Material 1

Supplementary Material 2

Supplementary Material 3

Supplementary Material 4

## Data Availability

The datasets used and/or analysed during the current study are available from the corresponding author on reasonable request.

## Abbreviations

AI: Artificial intelligence
CI: Confidence interval
DBC: Downs-and-Black checklist
FRE: Flesch Reading Ease Score
FKGL: Flesch Kincaid Grade Level
LLM: Large language model
LLM-DBC: Adaption of modified Downs-and-Black checklist by Zadro et al. for large language model studies
MD: Mean difference
n: Number
REML: Restricted Maximum Likelihood
