# Supplementary material for: Comparison of the readability of ChatGPT and Bard in medical communication: a meta-analysis
Source: BMC Med Inform Decis Mak. 2025 Sep 1;25:325. doi: 10.1186/s12911-025-03035-2 (PMC12403948; doi:10.1186/s12911-025-03035-2)
Supplement: Supplementary file 3 — Supplementary Material 3 [file 12911_2025_3035_MOESM3_ESM.docx]

**Meta-Analysis: Continuous Outcomes with Raw Data**

| **Notes** | | |
| --- | --- | --- |
| Output Created | | 11-JUL-2025 15:05:54 |
| Comments | |  |
| Input | Data | C:\Users\Bibliothek.MH-HANNOVER\Desktop\2025_04_02_llm_data_fin_simplification_2025_07_11.sav |
|  | Active Dataset | DataSet1 |
|  | Filter | <none> |
|  | Weight | <none> |
|  | Split File | <none> |
|  | N of Rows in Working Data File | 8 |
| Missing Value Handling | Definition of Missing | User-defined missing values are treated as missing. |
|  | Cases Used | Each statistic is based on all valid data for the available variable(s) used in computing the statistic. |
| Syntax | | META CONTINUOUS /DATA TREATMENT=N(ChatGPT_FRE_n) MEAN(ChatGPT_FRE_mean) STD(ChatGPT_FRE_sd) CONTROL=N(Bard_FRE_n) MEAN(Bard_FRE_mean) STD(Bard_FRE_sd) STUDY=FirstauthorYEAR ESTYPE=MEAN_DIFF(UNEQUAL) /CRITERIA CILEVEL=95 SCOPE=AVAILABLE CLASSMISSING=EXCLUDE MAXITER=100 MAXSTEP=5 CONVERGENCE=0.000001 /INFERENCE MODEL=RANDOM ESTIMATE=REML ADJUSTSE=KNAPP_HARTUNG /PRINT HOMOGENEITY HETEROGENEITY INDIVIDUAL PREDICTION /FORESTPLOT DISPLAY=ES SE CI PVAL WEIGHT POSITION=RIGHT ANNOTATIONS=HETEROGENEITY TEST REFLINES=OVERALL NULL /FUNNELPLOT YAXIS=SE LABEL=FirstauthorYEAR(AUTO). |
| Resources | Processor Time | 00:00:00,33 |
|  | Elapsed Time | 00:00:00,30 |

| **Meta-Analysis Summary** | |
| --- | --- |
| Data Type | Raw |
| Outcome Type | Continuous |
| Effect Size Measure | Mean Difference |
| Model | Random-effects |
| Weight | Inverse-variance^a^ |
| Estimation Method | REML |
| Standard Error Adjustment | Knapp-Hartung |
| a. Random-effects weights including both within- and between-study variance. | |

| **Case Processing Summary** | | |
| --- | --- | --- |
|  | N | Percent |
| Included | 3 | 37,5% |
| Missing | 5 | 62,5% |
| Invalid^a^ | 0 | 0,0% |
| Total | 8 | 100,0% |
| a. Nonpositive variance or standard error, or insufficient study size. | | |

| **Effect Size Estimates** | | | | | | | |
| --- | --- | --- | --- | --- | --- | --- | --- |
|  | Effect Size | Std. Error^a^ | t | Sig. (2-tailed) | 95% Confidence Interval | | 95% Prediction Interval^b^ |
|  |  |  |  |  | Lower | Upper | Lower |
| Overall | 5,026 | 5,8291 | ,862 | ,479 | -20,055 | 30,106 | -141,318 |

| **Effect Size Estimates** | | | | | | | | |
| --- | --- | --- | --- | --- | --- | --- | --- | --- |
|  | | | | 95% Prediction Interval | | | | |
|  |  |  |  | Upper | | | | |
| Overall | | | | 151,370 | | | | |
|  |  |  |  | |  |  |  |  |
|  |  |  |  | |  |  |  |  |

| a. Knapp-Hartung method is used for SE adjustment. |
| --- |
| b. Based on t-distribution. |

| **Effect Size Estimates for Individual Studies** | | | | | | |
| --- | --- | --- | --- | --- | --- | --- |
| Study | Effect Size | Std. Error^a^ | t | Sig. (2-tailed) | 95% Confidence Interval | |
|  |  |  |  |  | Lower | Upper |
| Rouhi2024 | 10,400 | 1,1646 | 8,930 | <,001 | 8,118 | 12,682 |
| Srinivasan2024_sim | 11,200 | 1,6286 | 6,877 | <,001 | 8,008 | 14,392 |
| Tepe2024 | -6,730 | 1,9639 | -3,427 | <,001 | -10,579 | -2,881 |

| **Effect Size Estimates for Individual Studies** | | | | | | | |
| --- | --- | --- | --- | --- | --- | --- | --- |
| Weight | | | | Weight (%) | | | |
|  |  |  |  |  |  |  |  |
| ,010 | | | | 33,8 | | | |
| ,010 | | | | 33,3 | | | |
| ,010 | | | | 32,9 | | | |
|  |  |  |  | |  |  |  |

| a. Knapp-Hartung method is used for SE adjustment. |
| --- |

| **Test of Homogeneity** | | | |
| --- | --- | --- | --- |
|  | Chi-square (Q statistic) | df | Sig. |
| Overall | 63,845 | 2 | <,001 |

| **Heterogeneity Measures** | | |
| --- | --- | --- |
| Overall | Tau-squared | 98,675 |
|  | H-squared | 41,725 |
|  | I-squared (%) | 97,6 |

**Meta-Analysis: Continuous Outcomes with Raw Data**

| **Notes** | | |
| --- | --- | --- |
| Output Created | | 11-JUL-2025 15:07:22 |
| Comments | |  |
| Input | Data | C:\Users\Bibliothek.MH-HANNOVER\Desktop\2025_04_02_llm_data_fin_simplification_2025_07_11.sav |
|  | Active Dataset | DataSet1 |
|  | Filter | <none> |
|  | Weight | <none> |
|  | Split File | <none> |
|  | N of Rows in Working Data File | 8 |
| Missing Value Handling | Definition of Missing | User-defined missing values are treated as missing. |
|  | Cases Used | Each statistic is based on all valid data for the available variable(s) used in computing the statistic. |
| Syntax | | META CONTINUOUS /DATA TREATMENT=N(ChatGPT_FKGL_n) MEAN(ChatGPT_FKGL_mean) STD(ChatGPT_FKGL_sd) CONTROL=N(Bard_FKGL_n) MEAN(Bard_FKGL_mean) STD(Bard_FKGL_sd) STUDY=FirstauthorYEAR ESTYPE=MEAN_DIFF(UNEQUAL) /CRITERIA CILEVEL=95 SCOPE=AVAILABLE CLASSMISSING=EXCLUDE MAXITER=100 MAXSTEP=5 CONVERGENCE=0.000001 /ANALYSIS SUBGROUP=COMPARISON /INFERENCE MODEL=RANDOM ESTIMATE=REML ADJUSTSE=KNAPP_HARTUNG /PRINT HOMOGENEITY HETEROGENEITY INDIVIDUAL PREDICTION /FORESTPLOT DISPLAY=ES SE CI PVAL WEIGHT POSITION=RIGHT ANNOTATIONS=HETEROGENEITY TEST REFLINES=OVERALL NULL /FUNNELPLOT YAXIS=SE LABEL=FirstauthorYEAR(AUTO). |
| Resources | Processor Time | 00:00:00,44 |
|  | Elapsed Time | 00:00:00,41 |

| **Meta-Analysis Summary** | |
| --- | --- |
| Data Type | Raw |
| Outcome Type | Continuous |
| Effect Size Measure | Mean Difference |
| Model | Random-effects |
| Weight | Inverse-variance^a^ |
| Estimation Method | REML |
| Standard Error Adjustment | Knapp-Hartung |
| Subgroup Analysis | COMPARISON |
| a. Random-effects weights including both within- and between-study variance. | |

| **Case Processing Summary** | | |
| --- | --- | --- |
|  | N | Percent |
| Included | 8 | 100,0% |
| Missing | 0 | 0,0% |
| Invalid^a^ | 0 | 0,0% |
| Total | 8 | 100,0% |
| a. Nonpositive variance or standard error, or insufficient study size. | | |

| **Effect Size Estimates for Subgroup Analysis** | | | | | | |
| --- | --- | --- | --- | --- | --- | --- |
|  | Effect Size | Std. Error^a^ | t | Sig. (2-tailed) | 95% Confidence Interval | |
|  |  |  |  |  | Lower | Upper |
| ChatGPT35_Bard^c^ | -1,000 | ,2469 | -4,050 | . | . | . |
| ChatGPT40_Bard | -1,681 | ,7545 | -2,228 | ,067 | -3,527 | ,165 |
| Overall | -1,593 | ,6581 | -2,420 | ,046 | -3,149 | -,036 |

| **Effect Size Estimates for Subgroup Analysis** | | | | | | | | |
| --- | --- | --- | --- | --- | --- | --- | --- | --- |
|  | | | 95% Prediction Interval^b^ | | | | | |
|  |  |  | Lower | | | Upper | | |
| ChatGPT35_Bard^c^ | | | . | | | . | | |
| ChatGPT40_Bard | | | -7,084 | | | 3,722 | | |
| Overall | | | -6,338 | | | 3,153 | | |
|  |  |  | |  |  | |  |  |
|  |  |  | |  |  | |  |  |
|  |  |  | |  |  | |  |  |

| a. Knapp-Hartung method is used for SE adjustment. |
| --- |
| b. Based on t-distribution. |
| c. Some statistics cannot be computed because this subgroup contains a single record. |

| **Effect Size Estimates for Individual Studies** | | | | | | |
| --- | --- | --- | --- | --- | --- | --- |
|  | Study | Effect Size | Std. Error^a^ | t | Sig. (2-tailed) | 95% Confidence Interval |
|  |  |  |  |  |  | Lower |
| ChatGPT35_Bard | Rouhi2024 | -1,000 | ,2469 | -4,050 | <,001 | -1,484 |
| ChatGPT40_Bard | Dihan2024b_sim | -,900 | ,4610 | -1,952 | ,051 | -1,803 |
|  | Dihan2024c_sim_A | -3,500 | ,3000 | -11,667 | <,001 | -4,088 |
|  | Dihan2024c_sim_B | ,240 | ,3130 | ,767 | ,443 | -,374 |
|  | Dihan2024d_sim | -3,700 | ,3354 | -11,031 | <,001 | -4,357 |
|  | Kianian2023_sim | -3,100 | ,6289 | -4,929 | <,001 | -4,333 |
|  | Srinivasan2024_sim | -2,300 | ,3484 | -6,602 | <,001 | -2,983 |
|  | Tepe2024 | 1,430 | ,3554 | 4,024 | <,001 | ,733 |

| **Effect Size Estimates for Individual Studies** | | | | | | | | | |
| --- | --- | --- | --- | --- | --- | --- | --- | --- | --- |
|  | | 95% Confidence Interval | | | Weight | | | Weight (%) | |
|  |  | Upper | | |  |  |  |  |  |
| ChatGPT35_Bard | | -,516 | | | ,295 | | | 12,8 | |
| ChatGPT40_Bard | | ,003 | | | ,282 | | | 12,3 | |
|  |  | -2,912 | | | ,293 | | | 12,7 | |
|  |  | ,854 | | | ,292 | | | 12,7 | |
|  |  | -3,043 | | | ,291 | | | 12,6 | |
|  |  | -1,867 | | | ,269 | | | 11,7 | |
|  |  | -1,617 | | | ,290 | | | 12,6 | |
|  |  | 2,127 | | | ,289 | | | 12,6 | |
|  |  | |  |  | |  |  | |  |

| a. Knapp-Hartung method is used for SE adjustment. |
| --- |

| **Test of Homogeneity** | | | |
| --- | --- | --- | --- |
|  | Chi-square (Q statistic) | df | Sig. |
| ChatGPT35_Bard^a^ | . | . | . |
| ChatGPT40_Bard | 198,379 | 6 | <,001 |
| Overall | 203,870 | 7 | <,001 |
| a. Statistics cannot be computed because this subgroup contains a single record. | | | |

| **Test of Subgroup Homogeneity** | | | |
| --- | --- | --- | --- |
|  | Chi-square (Q statistic) | df | Sig. |
| COMPARISON | ,732 | 1 | ,392 |

| **Heterogeneity Measures** | | |
| --- | --- | --- |
| ChatGPT35_Bard^a^ | Tau-squared | . |
|  | H-squared | . |
|  | I-squared (%) | . |
| ChatGPT40_Bard | Tau-squared | 3,849 |
|  | H-squared | 29,805 |
|  | I-squared (%) | 96,6 |
| Overall | Tau-squared | 3,329 |
|  | H-squared | 29,295 |
|  | I-squared (%) | 96,6 |
| a. Statistics cannot be computed because this subgroup contains insufficient records. | | |
