# Supplementary material for: Comparison of the readability of ChatGPT and Bard in medical communication: a meta-analysis
Source: BMC Med Inform Decis Mak. 2025 Sep 1;25:325. doi: 10.1186/s12911-025-03035-2 (PMC12403948; doi:10.1186/s12911-025-03035-2)
Supplement: Supplementary file 4 — Supplementary Material 4 [file 12911_2025_3035_MOESM4_ESM.docx]

| **Modified DBC*** | | **LLM-DBC** | |
| --- | --- | --- | --- |
| **Item** | **Scoring** | **Item** | **Scoring** |
| 1.Is the hypothesis/ aim/ objective of the study clearly described? | Yes or no (1,0) | 1. Is the objective of the study clear?   - If the hypothesis/ aim/ objective (e. g. difference in specific readability scores of texts generated or simplified by specific LLM-based chatbots) is not clearly described or explained in Abstract/ Introduction/ Methods, the question should be answered no. | Yes or no (1,0); 0, if unable to determine |
| 2. Are the main outcomes to be measured clearly described in the Introduction or Methods section?   - If the main outcomes are first mentioned in the Results section, the question should be answered no. | Yes or no (1,0) | 2. Are the main outcomes clearly described in the Introduction or Methods?   - If the main outcomes (e. g. specific readability scores for the analysis of texts generated or simplified by specific LLM-based chatbots) are not reported in Introduction/ Methods or not determined (e. g. via formulas or reference), the question should be answered no. | Yes or no (1,0); 0, if unable to determine |
| 3. Are the characteristics of the patients included in the study clearly described?   - In cohort studies and trials, inclusion and/or exclusion criteria should be given. In case-control studies, a case-definition and the source for controls should be given. | Yes or no (1,0) | 3. Are characteristics of the LLM & questions included in the study clearly described?   - If specific questions/ prompts (e. g. exact input and date) and specific LLM-based chatbots (e. g. exact model/ version/ settings and date) are not transparently reported, the question should be answered no. | Yes or no (1,0); 0, if unable to determine |
| 4. Are the main findings of the study clearly described?   - Simple outcome data (including denominators and numerators) should be reported for all major findings so that the reader can check the major analyses and conclusions. (This question does not cover statistical tests which are considered below). | Yes or no (1,0) | 4. Are the main findings of the study clearly described?   - If specific responses (e. g. exact output and date) and results of analysis are not transparently and scientific reported (e. g. international system of units or summary/ descriptive statistics)? | Yes or no (1,0); 0, if unable to determine |
| 5. Were the subjects asked to participate in the study representative of the entire population from which they were recruited?   - The study must identify the source population for patients and describe how the patients were selected. Patients would be representative if they comprised the entire source population, an unselected sample of consecutive patients, or a random sample. Random sampling is only feasible where a list of all members of the relevant. | Yes or no (1,0); 0 if unable to determine | 5. Were LLM & questions designed to participate in the study representative of the entire population from which they were recruited?   - If LLM-based chatbots and tasks/ queries/ prompts are not relevant or appropriate for this research field, the question should be answered no. The relevance of LLM-based chatbots and questions can be determined according published surveys, frequently asked questions from patients, guidelines from professional societies or from experience of data scientists/ medical experts. | Yes or no (1,0); 0, if unable to determine |
| 6. Were those subjects who were prepared to participate representative of the entire population from which they were recruited?   - The proportion of those asked who agreed should be stated. Validation that the sample was representative would include demonstrating that the distribution of the main confounding factors was the same in the study sample and the source population | Yes or no (1,0); 0, if unable to determine | 6. Were those LLM & questions who were prepared to participate representative and/ or relevant for this research field and study?   - If the selected/ specific LLM-based chatbots and selected/ specific tasks/ queries/ prompts are not representative for 5. or the information/ analysis is not available, the question should be answered no. | Yes or no (1,0); 0, if unable to determine |
| 7. Were the statistical tests used to assess the main outcomes appropriate?   - The statistical techniques used must be appropriate to the data. For example, nonparametric methods should be used for small sample sizes. Where little statistical analysis has been undertaken but where there is no evidence of bias, the question should be answered yes. If the distribution of the data (normal or not) is not described it must be assumed that the estimates used were appropriate and the question should be answered yes. | Yes or no (1,0); 0, if unable to determine | 7. Were the statistical tests used to assess main outcomes appropriate?   - If application and reporting of statistical analysis is not appropriate, the question should be answered no. For example, complete reporting of summary statistics, data distribution/ heteroskedasticity analysis and appropriate selection of tests for comparison or correlation analysis should be carried out and described in Methods/ Supplements. | Yes or no (1,0); 0, if unable to determine |
| 8. Were the main outcome measures used accurate (valid and reliable)?   - For studies where the outcome measures are clearly described, the question should be answered yes. For studies which refer to other work or that demonstrates the outcome measures are accurate, the question should be answered as yes. | Yes or no (1,0); 0, if unable to determine | 8. Were main outcome measures used accurate (valid and reliable)?   - If the main outcome measures (e. g. specific readability scores) are not accurate, the question should be answered no. Outcome measures should be objective, valid, reliable or reproducible, if possible. | Yes or no (1,0); 0, if unable to determine |

*In this table, the modified Downs-and-Black checklist by Zadro et al. and the adaption for studies on large language models is given. A maximum score of 8 points is possible and recommended. Assessment should be conducted for each individual outcome measure in a study. Abbreviations: DBC = Downs-and-Black checklist, LLM = large language models, LLM-DBC = adaption of modified DBC by Zadro et al. for studies on LLM.*

**References for modified DBC:*

- *Reference for original DBC: Downs SH, Black N. The feasibility of creating a checklist for the assessment of the methodological quality both of randomised and non-randomised studies of health care interventions. J Epidemiol Community Health. 1998;52(6):377-84*
- *Reference for modified DBC: Zadro J, et al. BMJ Open 2019; 9:e032329. doi: 10.1136/bmjopen-2019-032329*
