# Supplementary material for: Comparison of the readability of ChatGPT and Bard in medical communication: a meta-analysis
Source: BMC Med Inform Decis Mak. 2025 Sep 1;25:325. doi: 10.1186/s12911-025-03035-2 (PMC12403948; doi:10.1186/s12911-025-03035-2)
Supplement: Supplementary file 5 [file 12911_2025_3035_MOESM1_ESM.odt]

NEW_Systematic_Literature_Research

All Fields

(medicine) AND (ChatGPT) AND ((Bard) OR (Gemini)) AND ((flesch reading ease score) OR (flesch kincaid grade level))

Find publications on the comparison of ChatGPT and Bard/ Gemini using flesch reading ease score or flesch kincaid grade level in medicine! Find as many studies as possible!

GoogleScholar:390/ 17.01.25

(medicine) AND (ChatGPT) AND ((Bard) OR (Gemini)) AND ((flesch reading ease score) OR (flesch kincaid grade level))

Scopus:7/ 16.01.25

(medicine) AND (ChatGPT) AND ((Bard) OR (Gemini)) AND ((flesch reading ease score) OR (flesch kincaid grade level))

Scopus AI:7/16.01.25

Find publications on the comparison of ChatGPT and Bard/ Gemini using flesch reading ease score or flesch kincaid grade level in medicine! Find as many studies as possible!

PubMed:45/ 16.01.25

(medicine) AND (ChatGPT) AND ((Bard) OR (Gemini)) AND ((flesch reading ease score) OR (flesch kincaid grade level))

Ovid/Medline:31/ 16.01.25

(medicine and ChatGPT and (Bard or Gemini) and (flesch reading ease score or flesch kincaid grade level)).af.

CINAHL:0/ 16.01.25

medicine AND ChatGPT AND (Bard OR Gemini) AND (flesch reading ease score OR flesch kincaid grade level)

Web-Of-Science:31/ 16.01.25

(ALL=medicine) AND (ALL=ChatGPT) AND ((ALL=Bard) OR (ALL=Gemini)) AND ((ALL=flesch reading ease score) OR (ALL=flesch kincaid grade level))

Web-Of-Science Research Assistant: 8 -> 21/ 16.01.25

Find publications on the comparison of ChatGPT and Bard/ Gemini using flesch reading ease score or flesch kincaid grade level in medicine! Find as many studies as possible!

->

(chatgpt OR generative pre-trained transformer OR gpt) AND (bard OR gemini OR google ai chatbot) AND (flesch reading ease score OR flesch kincaid grade level OR readability tests) AND (medicine OR medical field OR healthcare) (Topic)
