# Supplementary material for: Comparison of the readability of ChatGPT and Bard in medical communication: a meta-analysis
Source: BMC Med Inform Decis Mak. 2025 Sep 1;25:325. doi: 10.1186/s12911-025-03035-2 (PMC12403948; doi:10.1186/s12911-025-03035-2)
Supplement: Supplementary file 6 [file 12911_2025_3035_MOESM2_ESM.odt]

Meta-Analysis: Continuous Outcomes with Raw Data

| Notes |
| --- |
| Output Created | 11-JUL-2025 15:16:51 |
| Comments |  |
| Input | Data | C:\Users\Bibliothek.MH-HANNOVER\Desktop\2025_04_02_llm_data_fin_generation_2025_05_30_corrected_2025_07_11.sav |
| Active Dataset | DataSet2 |
| Filter | <none> |
| Weight | <none> |
| Split File | <none> |
| N of Rows in Working Data File | 70 |
| Missing Value Handling | Definition of Missing | User-defined missing values are treated as missing. |
| Cases Used | Each statistic is based on all valid data for the available variable(s) used in computing the statistic. |
| Syntax | META CONTINUOUS /DATA TREATMENT=N(ChatGPT_FRE_n) MEAN(ChatGPT_FRE_mean) STD(ChatGPT_FRE_sd) CONTROL=N(Bard_FRE_n) MEAN(Bard_FRE_mean) STD(Bard_FRE_sd)   STUDY=FirstauthorYEAR ESTYPE=MEAN_DIFF(UNEQUAL) /CRITERIA CILEVEL=95 SCOPE=AVAILABLE CLASSMISSING=EXCLUDE MAXITER=100 MAXSTEP=5 CONVERGENCE=0.000001 /ANALYSIS SUBGROUP=COMPARISON /INFERENCE MODEL=RANDOM ESTIMATE=REML ADJUSTSE=KNAPP_HARTUNG /BIAS INTERCEPT=INCLUDE /PRINT  HOMOGENEITY HETEROGENEITY INDIVIDUAL PREDICTION /FORESTPLOT DISPLAY=ES SE CI PVAL WEIGHT POSITION=RIGHT ANNOTATIONS=HOMOGENEITY HETEROGENEITY TEST REFLINES=OVERALL NULL /FUNNELPLOT YAXIS=SE LABEL=FirstauthorYEAR(AUTO). |
| Resources | Processor Time | 00:00:01,36 |
| Elapsed Time | 00:00:00,62 |

[DataSet2] C:\Users\Bibliothek.MH-HANNOVER\Desktop\2025_04_02_llm_data_fin_generation_2025_05_30_corrected_2025_07_11.sav

| Meta-Analysis Summary |
| --- |
| Data Type | Raw |
| Outcome Type | Continuous |
| Effect Size Measure | Mean Difference |
| Model | Random-effects |
| Weight | Inverse-variancea |
| Estimation Method | REML |
| Standard Error Adjustment | Knapp-Hartung |
| Subgroup Analysis | COMPARISON |
| a. Random-effects weights including both within- and between-study variance. |

| Case Processing Summary |
| --- |
|  | N | Percent |
| Included | 43 | 61,4% |
| Missing | 27 | 38,6% |
| Invalida | 0 | 0,0% |
| Total | 70 | 100,0% |
| a. Nonpositive variance or standard error, or insufficient study size. |

| Effect Size Estimates for Subgroup Analysis |
| --- |
|  | Effect Size | Std. Errora | t | Sig. (2-tailed) | 95% Confidence Interval |
| Lower | Upper |
| ChatGPT35_Bard | -16,072 | 3,9013 | -4,120 | ,003 | -24,897 | -7,246 |
| ChatGPT35_Gemini | -4,510 | 1,8666 | -2,416 | ,039 | -8,732 | -,287 |
| ChatGPT40_Bard | -12,013 | 1,9104 | -6,288 | <,001 | -16,217 | -7,808 |
| ChatGPT40_Gemini | -7,913 | 1,6900 | -4,682 | <,001 | -11,678 | -4,147 |
| Overall | -10,360 | 1,3498 | -7,675 | <,001 | -13,083 | -7,636 |

| Effect Size Estimates for Subgroup Analysis |
| --- |
|  | 95% Prediction Intervalb |
| Lower | Upper |
| ChatGPT35_Bard | -45,030 | 12,886 |
| ChatGPT35_Gemini | -15,424 | 6,405 |
| ChatGPT40_Bard | -25,982 | 1,957 |
| ChatGPT40_Gemini | -18,628 | 2,803 |
| Overall | -27,254 | 6,535 |
|  |  |  |  |  |  |  |
|  |  |  |  |  |  |  |

| a. Knapp-Hartung method is used for SE adjustment. |
| --- |
| b. Based on t-distribution. |

| Effect Size Estimates for Individual Studies |
| --- |
|  | Study | Effect Size | Std. Errora | t | Sig. (2-tailed) | 95% Confidence Interval | Weight | Weight (%) |
| Lower | Upper |
| ChatGPT35_Bard | Carlson2024 | -19,270 | 3,2302 | -5,966 | <,001 | -25,601 | -12,939 | ,013 | 2,4 |
| Dogan2024 | -3,100 | 1,2132 | -2,555 | ,011 | -5,478 | -,722 | ,014 | 2,8 |
| Ichhpujani2024 | -35,400 | 1,9997 | -17,703 | <,001 | -39,319 | -31,481 | ,014 | 2,7 |
| Kattih2024 | 8,400 | 3,7312 | 2,251 | ,024 | 1,087 | 15,713 | ,012 | 2,3 |
| Lauck2024 | -19,700 | 2,5116 | -7,843 | <,001 | -24,623 | -14,777 | ,013 | 2,6 |
| Lee2025z | -16,650 | 2,9138 | -5,714 | <,001 | -22,361 | -10,939 | ,013 | 2,5 |
| Nichani2024 | -16,800 | 7,1858 | -2,338 | ,019 | -30,884 | -2,716 | ,008 | 1,6 |
| Patnaik2024 | -25,400 | 4,2068 | -6,038 | <,001 | -33,645 | -17,155 | ,012 | 2,2 |
| Seth2023 | -9,790 | 8,1921 | -1,195 | ,232 | -25,846 | 6,266 | ,007 | 1,4 |
| Yilmaz2024 | -21,120 | 2,8894 | -7,309 | <,001 | -26,783 | -15,457 | ,013 | 2,5 |
| ChatGPT35_Gemini | Arca2024 | -5,200 | 1,8518 | -2,808 | ,005 | -8,829 | -1,571 | ,014 | 2,7 |
| Arora2024 | -21,730 | 7,1873 | -3,023 | ,002 | -35,817 | -7,643 | ,008 | 1,6 |
| Cao2024 | -1,000 | 1,4606 | -,685 | ,494 | -3,863 | 1,863 | ,014 | 2,7 |
| ELSenbawy2025 | -2,370 | 13,6554 | -,174 | ,862 | -29,134 | 24,394 | ,004 | ,8 |
| Hancı2024 | -2,220 | 1,0409 | -2,133 | ,033 | -4,260 | -,180 | ,014 | 2,8 |
| Joseph2024 | -2,160 | 8,0694 | -,268 | ,789 | -17,976 | 13,656 | ,008 | 1,4 |
| Karnan2024 | -10,470 | 4,9579 | -2,112 | ,035 | -20,187 | -,753 | ,011 | 2,1 |
| Lee2024 | ,700 | 1,6928 | ,414 | ,679 | -2,618 | 4,018 | ,014 | 2,7 |
| Lim2024 | -,640 | 3,1790 | -,201 | ,840 | -6,871 | 5,591 | ,013 | 2,5 |
| Sonmezoglu2024 | -12,020 | 2,8087 | -4,279 | <,001 | -17,525 | -6,515 | ,013 | 2,5 |
| ChatGPT40_Bard | Alasker2024 | -13,800 | 1,9922 | -6,927 | <,001 | -17,705 | -9,895 | ,014 | 2,7 |
| Connors2024 | -24,140 | 3,2557 | -7,415 | <,001 | -30,521 | -17,759 | ,013 | 2,4 |
| Lee2024c | -21,210 | ,8160 | -25,993 | <,001 | -22,809 | -19,611 | ,015 | 2,8 |
| Lim2023 | -5,160 | 5,4301 | -,950 | ,342 | -15,803 | 5,483 | ,010 | 2,0 |
| Lim2024b | -9,400 | 3,1689 | -2,966 | ,003 | -15,611 | -3,189 | ,013 | 2,5 |
| Rokhshad2025 | -16,200 | 2,8778 | -5,629 | <,001 | -21,840 | -10,560 | ,013 | 2,5 |
| San2024_gen_A | -11,840 | 2,2184 | -5,337 | <,001 | -16,188 | -7,492 | ,014 | 2,6 |
| San2024_gen_B | -7,560 | 2,3366 | -3,235 | ,001 | -12,140 | -2,980 | ,014 | 2,6 |
| San2024_gen_C | -11,220 | 2,3690 | -4,736 | <,001 | -15,863 | -6,577 | ,014 | 2,6 |
| San2024_gen_D | -2,930 | 2,4996 | -1,172 | ,241 | -7,829 | 1,969 | ,013 | 2,6 |
| Srinivasan2024_gen | -13,600 | 1,8613 | -7,307 | <,001 | -17,248 | -9,952 | ,014 | 2,7 |
| Xie2023 | -2,700 | 3,8127 | -,708 | ,479 | -10,173 | 4,773 | ,012 | 2,3 |
| ChatGPT40_Gemini | Adithya2024 | -8,740 | 9,3641 | -,933 | ,351 | -27,093 | 9,613 | ,006 | 1,2 |
| Aydın2024 | -5,600 | 4,4092 | -1,270 | ,204 | -14,242 | 3,042 | ,011 | 2,2 |
| Gunesli2024 | -15,200 | 2,2837 | -6,656 | <,001 | -19,676 | -10,724 | ,014 | 2,6 |
| Guven2024 | -5,490 | 1,5988 | -3,434 | <,001 | -8,624 | -2,356 | ,014 | 2,7 |
| Lim2024c | 3,980 | 4,7380 | ,840 | ,401 | -5,306 | 13,266 | ,011 | 2,1 |
| Meyer2024 | -4,200 | 5,3712 | -,782 | ,434 | -14,727 | 6,327 | ,010 | 2,0 |
| Nian2025 | -11,000 | 3,8761 | -2,838 | ,005 | -18,597 | -3,403 | ,012 | 2,3 |
| Nian2025b | -10,100 | 3,5511 | -2,844 | ,004 | -17,060 | -3,140 | ,012 | 2,4 |
| Reyhan2024 | -5,850 | 2,7249 | -2,147 | ,032 | -11,191 | -,509 | ,013 | 2,5 |
| Rocha-Silva2025 | -3,480 | 4,0892 | -,851 | ,395 | -11,495 | 4,535 | ,012 | 2,3 |
| Tepe2024a | -15,300 | 2,8246 | -5,417 | <,001 | -20,836 | -9,764 | ,013 | 2,5 |
| a. Knapp-Hartung method is used for SE adjustment. |

| Test of Homogeneity |
| --- |
|  | Chi-square (Q statistic) | df | Sig. |
| ChatGPT35_Bard | 253,631 | 9 | <,001 |
| ChatGPT35_Gemini | 28,325 | 9 | <,001 |
| ChatGPT40_Bard | 118,370 | 11 | <,001 |
| ChatGPT40_Gemini | 28,977 | 10 | ,001 |
| Overall | 691,497 | 42 | <,001 |

| Test of Subgroup Homogeneity |
| --- |
|  | Chi-square (Q statistic) | df | Sig. |
| COMPARISON | 12,129 | 3 | ,007 |

| Heterogeneity Measures |
| --- |
| ChatGPT35_Bard | Tau-squared | 142,475 |
| H-squared | 20,123 |
| I-squared (%) | 95,0 |
| ChatGPT35_Gemini | Tau-squared | 18,918 |
| H-squared | 4,742 |
| I-squared (%) | 78,9 |
| ChatGPT40_Bard | Tau-squared | 35,660 |
| H-squared | 8,703 |
| I-squared (%) | 88,5 |
| ChatGPT40_Gemini | Tau-squared | 19,583 |
| H-squared | 2,931 |
| I-squared (%) | 65,9 |
| Overall | Tau-squared | 68,157 |
| H-squared | 13,543 |
| I-squared (%) | 92,6 |

| Egger's Regression-Based Testa |
| --- |
|  | Parameter | Coefficient | Std. Error | t | Sig. (2-tailed) | 95% Confidence Interval |
| Lower |
| ChatGPT35_Bard | (Intercept) | -18,542 | 8,5304 | -2,174 | ,061 | -38,213 |
| SEb | ,691 | 2,0814 | ,332 | ,748 | -4,108 |
| ChatGPT35_Gemini | (Intercept) | -1,199 | 2,6654 | -,450 | ,665 | -7,346 |
| SEb | -1,161 | ,8138 | -1,426 | ,192 | -3,037 |
| ChatGPT40_Bard | (Intercept) | -19,326 | 4,8450 | -3,989 | ,003 | -30,122 |
| SEb | 2,840 | 1,7630 | 1,611 | ,138 | -1,088 |
| ChatGPT40_Gemini | (Intercept) | -11,777 | 4,2739 | -2,755 | ,022 | -21,445 |
| SEb | 1,117 | 1,1366 | ,982 | ,352 | -1,454 |
| Overall | (Intercept) | -11,485 | 2,6517 | -4,331 | <,001 | -16,840 |
| SEb | ,345 | ,6956 | ,495 | ,623 | -1,060 |

| Egger's Regression-Based Testa |
| --- |
|  | Parameter | 95% Confidence Interval |
| Upper |
| ChatGPT35_Bard | (Intercept) | 1,129 |
| SEb | 5,491 |
| ChatGPT35_Gemini | (Intercept) | 4,947 |
| SEb | ,716 |
| ChatGPT40_Bard | (Intercept) | -8,531 |
| SEb | 6,769 |
| ChatGPT40_Gemini | (Intercept) | -2,108 |
| SEb | 3,688 |
| Overall | (Intercept) | -6,130 |
| SEb | 1,749 |
|  |  |  |  |  |  |  |
|  |  |  |  |  |  |  |

| a. Random effects meta-regression with the Knapp-Hartung SE adjustment. |
| --- |
| b. Standard error of effect size |

img.emf

img.emf

img.emf

Meta-Analysis: Continuous Outcomes with Raw Data

| Notes |
| --- |
| Output Created | 11-JUL-2025 15:20:40 |
| Comments |  |
| Input | Data | C:\Users\Bibliothek.MH-HANNOVER\Desktop\2025_04_02_llm_data_fin_generation_2025_05_30_corrected_2025_07_11.sav |
| Active Dataset | DataSet2 |
| Filter | <none> |
| Weight | <none> |
| Split File | <none> |
| N of Rows in Working Data File | 70 |
| Missing Value Handling | Definition of Missing | User-defined missing values are treated as missing. |
| Cases Used | Each statistic is based on all valid data for the available variable(s) used in computing the statistic. |
| Syntax | META CONTINUOUS /DATA TREATMENT=N(ChatGPT_FKGL_n) MEAN(ChatGPT_FKGL_mean) STD(ChatGPT_FKGL_sd) CONTROL=N(Bard_FKGL_n) MEAN(Bard_FKGL_mean) STD(Bard_FKGL_sd)   STUDY=FirstauthorYEAR ESTYPE=MEAN_DIFF(UNEQUAL) /CRITERIA CILEVEL=95 SCOPE=AVAILABLE CLASSMISSING=EXCLUDE MAXITER=100 MAXSTEP=5 CONVERGENCE=0.000001 /ANALYSIS SUBGROUP=COMPARISON /INFERENCE MODEL=RANDOM ESTIMATE=REML ADJUSTSE=KNAPP_HARTUNG /BIAS INTERCEPT=INCLUDE /PRINT  HOMOGENEITY HETEROGENEITY INDIVIDUAL PREDICTION /FORESTPLOT DISPLAY=ES SE CI PVAL WEIGHT POSITION=RIGHT ANNOTATIONS=HOMOGENEITY HETEROGENEITY TEST REFLINES=OVERALL NULL /FUNNELPLOT YAXIS=SE LABEL=FirstauthorYEAR(AUTO). |
| Resources | Processor Time | 00:00:00,56 |
| Elapsed Time | 00:00:00,44 |

| Meta-Analysis Summary |
| --- |
| Data Type | Raw |
| Outcome Type | Continuous |
| Effect Size Measure | Mean Difference |
| Model | Random-effects |
| Weight | Inverse-variancea |
| Estimation Method | REML |
| Standard Error Adjustment | Knapp-Hartung |
| Subgroup Analysis | COMPARISON |
| a. Random-effects weights including both within- and between-study variance. |

| Case Processing Summary |
| --- |
|  | N | Percent |
| Included | 66 | 94,3% |
| Missing | 4 | 5,7% |
| Invalida | 0 | 0,0% |
| Total | 70 | 100,0% |
| a. Nonpositive variance or standard error, or insufficient study size. |

| Effect Size Estimates for Subgroup Analysis |
| --- |
|  | Effect Size | Std. Errora | t | Sig. (2-tailed) | 95% Confidence Interval |
| Lower | Upper |
| ChatGPT35_Bard | 2,852 | ,4166 | 6,846 | <,001 | 1,977 | 3,727 |
| ChatGPT35_Gemini | 1,215 | ,3222 | 3,770 | ,004 | ,497 | 1,933 |
| ChatGPT40_Bard | ,643 | ,5296 | 1,213 | ,238 | -,456 | 1,741 |
| ChatGPT40_Gemini | 1,952 | ,4163 | 4,690 | <,001 | 1,045 | 2,860 |
| Overall | 1,617 | ,2645 | 6,116 | <,001 | 1,089 | 2,145 |

| Effect Size Estimates for Subgroup Analysis |
| --- |
|  | 95% Prediction Intervalb |
| Lower | Upper |
| ChatGPT35_Bard | -,870 | 6,575 |
| ChatGPT35_Gemini | -1,102 | 3,532 |
| ChatGPT40_Bard | -4,710 | 5,995 |
| ChatGPT40_Gemini | -1,207 | 5,112 |
| Overall | -2,607 | 5,842 |
|  |  |  |  |  |  |  |
|  |  |  |  |  |  |  |

| a. Knapp-Hartung method is used for SE adjustment. |
| --- |
| b. Based on t-distribution. |

| Effect Size Estimates for Individual Studies |
| --- |
|  | Study | Effect Size | Std. Errora | t | Sig. (2-tailed) | 95% Confidence Interval | Weight | Weight (%) |
| Lower | Upper |
| ChatGPT35_Bard | Carlson2024 | 4,130 | ,5588 | 7,391 | <,001 | 3,035 | 5,225 | ,212 | 1,6 |
| Garg2024_gen_A | 4,510 | ,3844 | 11,734 | <,001 | 3,757 | 5,263 | ,220 | 1,6 |
| Garg2024_gen_B | 3,220 | ,4261 | 7,556 | <,001 | 2,385 | 4,055 | ,218 | 1,6 |
| Garg2024_gen_C | ,910 | ,4194 | 2,170 | ,030 | ,088 | 1,732 | ,218 | 1,6 |
| Garg2024_gen_D | 3,990 | ,4041 | 9,874 | <,001 | 3,198 | 4,782 | ,219 | 1,6 |
| Ichhpujani2024 | 6,460 | ,4327 | 14,931 | <,001 | 5,612 | 7,308 | ,218 | 1,6 |
| Kattih2024 | -,600 | ,5075 | -1,182 | ,237 | -1,595 | ,395 | ,215 | 1,6 |
| Lauck2024 | 2,900 | ,5605 | 5,174 | <,001 | 1,801 | 3,999 | ,212 | 1,6 |
| Lee2025z | 3,330 | ,5746 | 5,796 | <,001 | 2,204 | 4,456 | ,211 | 1,6 |
| Nichani2024 | 4,200 | 1,5165 | 2,770 | ,006 | 1,228 | 7,172 | ,149 | 1,1 |
| Patnaik2024 | 5,300 | ,5849 | 9,061 | <,001 | 4,154 | 6,446 | ,211 | 1,6 |
| Seth2023 | ,440 | 1,4787 | ,298 | ,766 | -2,458 | 3,338 | ,152 | 1,1 |
| Warren2024_gen_A | 3,800 | ,9220 | 4,122 | <,001 | 1,993 | 5,607 | ,190 | 1,4 |
| Warren2024_gen_B | 1,400 | ,6146 | 2,278 | ,023 | ,195 | 2,605 | ,209 | 1,5 |
| Warren2025_gen_A | 1,500 | ,4397 | 3,411 | <,001 | ,638 | 2,362 | ,218 | 1,6 |
| Warren2025_gen_B | 1,100 | ,5686 | 1,934 | ,053 | -,014 | 2,214 | ,212 | 1,6 |
| Warren2025_gen_C | 2,600 | ,9343 | 2,783 | ,005 | ,769 | 4,431 | ,190 | 1,4 |
| Wu2024 | 2,600 | 1,5945 | 1,631 | ,103 | -,525 | 5,725 | ,144 | 1,1 |
| Yau2024 | 1,850 | ,7147 | 2,589 | ,010 | ,449 | 3,251 | ,204 | 1,5 |
| ChatGPT35_Gemini | Arca2024 | 1,760 | ,2754 | 6,391 | <,001 | 1,220 | 2,300 | ,223 | 1,6 |
| Arora2024 | 1,370 | 2,9624 | ,462 | ,644 | -4,436 | 7,176 | ,076 | ,6 |
| Cao2024 | 1,000 | ,3651 | 2,739 | ,006 | ,284 | 1,716 | ,221 | 1,6 |
| ELSenbawy2025 | ,900 | 2,2169 | ,406 | ,685 | -3,445 | 5,245 | ,107 | ,8 |
| Hancı2024 | -,130 | ,1574 | -,826 | ,409 | -,439 | ,179 | ,226 | 1,7 |
| Joseph2024 | 2,590 | 1,5674 | 1,652 | ,098 | -,482 | 5,662 | ,146 | 1,1 |
| Karnan2024 | 2,670 | 1,4133 | 1,889 | ,059 | -,100 | 5,440 | ,156 | 1,2 |
| Lee2024 | -,320 | ,2806 | -1,141 | ,254 | -,870 | ,230 | ,223 | 1,6 |
| Lee2024b | 2,420 | ,4014 | 6,029 | <,001 | 1,633 | 3,207 | ,219 | 1,6 |
| Lim2024 | 1,270 | ,6338 | 2,004 | ,045 | ,028 | 2,512 | ,208 | 1,5 |
| Sonmezoglu2024 | 1,940 | ,5932 | 3,271 | ,001 | ,777 | 3,103 | ,210 | 1,6 |
| ChatGPT40_Bard | Acharya2024 | -,400 | ,4663 | -,858 | ,391 | -1,314 | ,514 | ,217 | 1,6 |
| Alasker2024 | 2,230 | ,3876 | 5,753 | <,001 | 1,470 | 2,990 | ,220 | 1,6 |
| Azzopardi2024 | -2,270 | ,2130 | -10,658 | <,001 | -2,687 | -1,853 | ,225 | 1,7 |
| Dihan2024b_gen_A | -,800 | ,3202 | -2,499 | ,012 | -1,427 | -,173 | ,222 | 1,6 |
| Dihan2024b_gen_B | -1,400 | ,2062 | -6,791 | <,001 | -1,804 | -,996 | ,225 | 1,7 |
| Dihan2024c_gen_A | ,330 | ,1897 | 1,739 | ,082 | -,042 | ,702 | ,225 | 1,7 |
| Dihan2024c_gen_B | -3,700 | ,2214 | -16,715 | <,001 | -4,134 | -3,266 | ,225 | 1,7 |
| Dihan2024d_gen_A | ,300 | ,2110 | 1,422 | ,155 | -,113 | ,713 | ,225 | 1,7 |
| Dihan2024d_gen_B | -3,300 | ,1897 | -17,393 | <,001 | -3,672 | -2,928 | ,225 | 1,7 |
| Kianian2023_gen_A | -4,200 | ,4561 | -9,209 | <,001 | -5,094 | -3,306 | ,217 | 1,6 |
| Kianian2023_gen_B | -1,600 | ,4669 | -3,427 | <,001 | -2,515 | -,685 | ,216 | 1,6 |
| Lee2024c | 3,000 | ,1210 | 24,786 | <,001 | 2,763 | 3,237 | ,226 | 1,7 |
| Lim2023 | ,860 | ,8731 | ,985 | ,325 | -,851 | 2,571 | ,194 | 1,4 |
| Lim2024b | 2,000 | ,5553 | 3,602 | <,001 | ,912 | 3,088 | ,212 | 1,6 |
| Ocakoglu2023 | 4,160 | ,4474 | 9,298 | <,001 | 3,283 | 5,037 | ,217 | 1,6 |
| Rokhshad2025 | 2,500 | ,5025 | 4,975 | <,001 | 1,515 | 3,485 | ,215 | 1,6 |
| San2024_gen_A | 3,080 | ,3626 | 8,494 | <,001 | 2,369 | 3,791 | ,221 | 1,6 |
| San2024_gen_B | 2,230 | ,4080 | 5,465 | <,001 | 1,430 | 3,030 | ,219 | 1,6 |
| San2024_gen_C | 2,900 | ,3851 | 7,530 | <,001 | 2,145 | 3,655 | ,220 | 1,6 |
| San2024_gen_D | 1,370 | ,4211 | 3,253 | ,001 | ,545 | 2,195 | ,218 | 1,6 |
| Srinivasan2024_gen | 2,000 | ,4038 | 4,953 | <,001 | 1,209 | 2,791 | ,219 | 1,6 |
| Xie2023 | ,500 | ,7095 | ,705 | ,481 | -,891 | 1,891 | ,204 | 1,5 |
| Yalla2024 | 5,110 | ,0943 | 54,182 | <,001 | 4,925 | 5,295 | ,227 | 1,7 |
| ChatGPT40_Gemini | Adithya2024 | ,280 | 1,5073 | ,186 | ,853 | -2,674 | 3,234 | ,150 | 1,1 |
| Aydın2024 | 1,730 | ,6555 | 2,639 | ,008 | ,445 | 3,015 | ,207 | 1,5 |
| Giammanco2025 | 2,600 | ,8274 | 3,143 | ,002 | ,978 | 4,222 | ,197 | 1,5 |
| Gunesli2024 | 3,300 | ,4170 | 7,914 | <,001 | 2,483 | 4,117 | ,219 | 1,6 |
| Guven2024 | 1,950 | ,2919 | 6,681 | <,001 | 1,378 | 2,522 | ,223 | 1,6 |
| Li2025 | -,130 | ,2259 | -,576 | ,565 | -,573 | ,313 | ,225 | 1,7 |
| Lim2024c | -,180 | 1,0859 | -,166 | ,868 | -2,308 | 1,948 | ,179 | 1,3 |
| Nian2025 | 3,400 | ,6200 | 5,484 | <,001 | 2,185 | 4,615 | ,209 | 1,5 |
| Nian2025b | 2,100 | ,6325 | 3,320 | <,001 | ,860 | 3,340 | ,208 | 1,5 |
| Reyhan2024 | 2,180 | ,5424 | 4,020 | <,001 | 1,117 | 3,243 | ,213 | 1,6 |
| Rocha-Silva2025 | -,480 | ,6211 | -,773 | ,440 | -1,697 | ,737 | ,209 | 1,5 |
| Tepe2024a | 3,630 | ,5686 | 6,384 | <,001 | 2,516 | 4,744 | ,212 | 1,6 |
| Zhao2024 | 3,620 | ,1789 | 20,235 | <,001 | 3,269 | 3,971 | ,226 | 1,7 |
| a. Knapp-Hartung method is used for SE adjustment. |

| Test of Homogeneity |
| --- |
|  | Chi-square (Q statistic) | df | Sig. |
| ChatGPT35_Bard | 217,502 | 18 | <,001 |
| ChatGPT35_Gemini | 81,628 | 10 | <,001 |
| ChatGPT40_Bard | 3740,944 | 22 | <,001 |
| ChatGPT40_Gemini | 212,990 | 12 | <,001 |
| Overall | 4499,225 | 65 | <,001 |

| Test of Subgroup Homogeneity |
| --- |
|  | Chi-square (Q statistic) | df | Sig. |
| COMPARISON | 13,245 | 3 | ,004 |

| Heterogeneity Measures |
| --- |
| ChatGPT35_Bard | Tau-squared | 2,939 |
| H-squared | 10,414 |
| I-squared (%) | 90,4 |
| ChatGPT35_Gemini | Tau-squared | ,945 |
| H-squared | 6,913 |
| I-squared (%) | 85,5 |
| ChatGPT40_Bard | Tau-squared | 6,344 |
| H-squared | 101,906 |
| I-squared (%) | 99,0 |
| ChatGPT40_Gemini | Tau-squared | 1,887 |
| H-squared | 11,938 |
| I-squared (%) | 91,6 |
| Overall | Tau-squared | 4,401 |
| H-squared | 41,594 |
| I-squared (%) | 97,6 |

| Egger's Regression-Based Testa |
| --- |
|  | Parameter | Coefficient | Std. Error | t | Sig. (2-tailed) | 95% Confidence Interval |
| Lower |
| ChatGPT35_Bard | (Intercept) | 3,220 | ,9424 | 3,417 | ,003 | 1,232 |
| SEb | -,560 | 1,2768 | -,439 | ,666 | -3,254 |
| ChatGPT35_Gemini | (Intercept) | ,785 | ,4538 | 1,729 | ,118 | -,242 |
| SEb | ,780 | ,6175 | 1,264 | ,238 | -,617 |
| ChatGPT40_Bard | (Intercept) | ,251 | 1,2381 | ,203 | ,841 | -2,324 |
| SEb | 1,058 | 3,0087 | ,352 | ,729 | -5,199 |
| ChatGPT40_Gemini | (Intercept) | 2,794 | ,8753 | 3,192 | ,009 | ,868 |
| SEb | -1,480 | 1,3551 | -1,093 | ,298 | -4,463 |
| Overall | (Intercept) | 1,278 | ,4496 | 2,843 | ,006 | ,380 |
| SEb | ,612 | ,6558 | ,933 | ,354 | -,698 |

| Egger's Regression-Based Testa |
| --- |
|  | Parameter | 95% Confidence Interval |
| Upper |
| ChatGPT35_Bard | (Intercept) | 5,209 |
| SEb | 2,133 |
| ChatGPT35_Gemini | (Intercept) | 1,811 |
| SEb | 2,177 |
| ChatGPT40_Bard | (Intercept) | 2,826 |
| SEb | 7,315 |
| ChatGPT40_Gemini | (Intercept) | 4,721 |
| SEb | 1,502 |
| Overall | (Intercept) | 2,176 |
| SEb | 1,922 |
|  |  |  |  |  |  |  |
|  |  |  |  |  |  |  |

| a. Random effects meta-regression with the Knapp-Hartung SE adjustment. |
| --- |
| b. Standard error of effect size |

img.emf

img.emf

img.emf
